# Enhancing early bonding between mothers and preterm infants: findings from a Multicentre Qualitative Study of Nurses *

**DOI:** 10.1590/1518-8345.7034.4200

**Published:** 2024-09-23

**Authors:** Nopi Nur Khasanah, Yeni Rustina, Dessie Wanda, Iskim Luthfa

**Affiliations:** 1 Sultan Agung Islamic University, College of Nursing, Semarang, Indonesia, Asia; 2 University of Indonesia, College of Nursing, Depok, Indonesia, Asia; 3 Scholarship holder at the Lembaga Pengelola Dana Pendidikan (LPDP), Indonesia

**Keywords:** Family Relations, City Hospitals, Infant Welfare, Mothers, Mother-Child Relations, Neonatal Nurses

## Abstract

**Objectives:**

this study aims to explore neonatal nurses’ experiences of facilitating early bonding between mothers and premature babies

**Method:**

a descriptive qualitative approach was adopted, using focus group discussions with 13 participants from four referral hospitals in a major city in a developing country. This was followed by in-depth interviews with three participants

**Results:**

three main themes emerged, highlighting the barriers and facilitators to early bonding between mothers and preterm infants. The study also explored care practices aimed at facilitating early bonding within the social environment of preterm infants, involving mothers, families, nurses and hospitals.

**Conclusions:**

the barriers and facilitators identified inform the development of intervention strategies for neonatal nurses to promote early bonding. Optimal early bonding requires enhanced nurse management skills, adequate hospital infrastructure and involvement of the patient’s family. These findings contribute to the advancement of health and nursing knowledge.

## Highlights

**(1)** Early bonding thrives with support from nurses, hospital and family alike.

**(2)** Prioritize stabilizing the mother’s condition before promoting mother-infant bonding.

**(3)** Neonatal nurses play a key role in promoting early mother-infant bonding.

**(4)** Comprehensive interventions should be adapted to the cultural norms of the hospital.

**(5)** Involvement of fathers can help nurses overcome various barriers to maternal care.

## Introduction

 The introduction of developmental care in neonatal units marks a significant shift in nursing practice by emphasizing the provision of appropriate stimulation for the development of premature infants ^( 1 )^ . While nurses can provide stimulation, it is optimal for preterm infants to receive it directly from their mothers. In addition to promoting optimal growth and development, maternal stimulation strengthens the bond between mother and preterm baby. Unfortunately, neonatal nurses often overlook working with parents and focus solely on the medical needs of the infant, neglecting to promote attachment between mothers and premature babies due to limited support for bonding ^( 2 )^ . However, neonatal nurses have a valuable opportunity to facilitate bonding ^( 3 )^ between mothers and their preterm infants ^( 4 )^ . Research suggests that early bonding between mother and premature baby has positive effects while the baby is still in the NICU ^( 5 )^ . 

 Neonatal nurses need to have a nuanced understanding of the different strategies for promoting bonding between mothers and their premature babies. This understanding is essential for the implementation of the family-centred care (FCC) approach to neonatal care. Nurses have a key role to play in fostering this bond, as preterm birth can challenge a woman’s perception of motherhood and leave her feeling inadequate to care for or protect her infant. Such feelings can hinder the development of a strong bond between mothers and preterm infants ^( 6 )^ . In addition, initiatives to involve parents in the NICU should be culturally sensitive to reduce bonding complications. Each mother’s needs vary according to her individual circumstances ^( 7 )^ . Those whose infants require NICU care often need emotional support from healthcare providers, clear and comprehensive information about their infants’ medical care, opportunities to participate in decision making, and involvement in infant care activities ^( 8 )^ . 

 Research on the role of nurses in the NICU environment is still limited, including research on facilitating early bonding between mothers and preterm infants. Needs assessment studies of NICUs ^( 9 )^ have identified an important role for neonatal nurses in meeting the needs of parents when their infants are in intensive care and their role in the health of preterm infants. The neonatal intensive care unit (NICU) is a therapeutic environment designed to care for high-risk neonates. The unstable condition of the infant, risky procedures and verbal harassment by parents are concerns faced by NICU nurses ^( 10 )^ . Therefore, this study aims to explore nurses’ experiences in facilitating bonding between mothers and their preterm infants in the NICU. 

## Method

### Study design

 This is a qualitative descriptive study with an interpretive approach ^( 11 )^ . This interpretive approach can explore and describe research problems and summarize a phenomenon comprehensively ^( 12 )^ . In addition, the research team has a common interest in the health and well-being of premature babies and consists of three women who are experts in paediatric nursing and one man who is an expert in family nursing. The research team does not guarantee a special relationship with any of the participants in this study. 

### Setting and participants

We used the focus group discussion (FGD) technique, followed by in-depth interviews to corroborate the findings. Data were collected through the Zoom meeting application using the FGD technique and face-to-face in-depth interview sessions. FGD activities were conducted with the main researcher and the family nurse. The FGD meeting took place twice. The first meeting was held in the afternoon because its online nature allowed participants working in different shifts (morning, afternoon and night) to participate. By mutual agreement, the second meeting was also held in the afternoon, as most of the nurses who are team leaders work on the morning shift. All nurses participated in the first FGD meeting on the same day and at the same time using the Zoom meeting application, and only two nurses did not participate in the second FGD meeting. Although there were network problems during the FGD activities, the participants answered all the questions. Meanwhile, in-depth interviews were conducted only by the main researcher. The interviews were conducted face-to-face in the training room of the neonatal unit immediately after the morning shift. The coding was done by the main researcher and the coding results were analysed by experts in paediatric nursing. The population in this study were nurses working in the NICU from September to December 2022.

 Participants were purposively selected using strategic critical case sampling ^( 13 )^ to maximise the information needed. The researchers explained the reasons for the research and arranged the participants’ routines for 2-3 hours/day for three months to build familiarity with four referral hospitals in a major city in a developing country. The hospitals were selected according to the following criteria: (1) public hospitals with a neonatal unit, (2) nationally accredited hospital, and (3) the neonatal unit was implementing FCC. In addition, participants had to meet the inclusion criteria of having worked in the NICU for at least five years, being a team leader, and being able to communicate online. There are only 2-3 team leaders in each hospital NICU. There were no dropouts as all participants were cooperative during the data collection process. 

### Data collection

The instrument used in this study was a general guide of questions related to nurses’ experiences in facilitating bonding between mothers and their premature babies. The questions were then developed according to the participants’ responses during the FGD and in-depth interviews. Several support tools were used during data collection, including stationery and field note sheets to record important details such as participants’ facial expressions and attitudes towards the researchers during data collection. In this study, a voice recorder was used during the in-depth interviews with three participants. However, it was not needed during the FGD activities as these were recorded using the Zoom meeting application. Prior to data collection, the researchers piloted the data collection tools by conducting FGDs with four nurses who were managers of the NICU rooms in each hospital where the study was conducted and conducting in-depth interviews with two nurses. The results of the FGDs and in-depth interviews were evaluated by experts in paediatric nursing to obtain feedback.

 The first step in the data collection process was to identify the potential participants based on the inclusion criteria set by the main researcher. The NICU manager assisted the main researcher by informing some nurses who met the criteria and were potential participants. The main researcher conducted an orientation session with the potential participants to build trust with them ^( 14 )^ . All potential participants were willing to sign the consent form for this research after the main researcher explained the aims, objectives and procedures to be followed during the research. Data collection using the FGD technique was conducted online in a Zoom room to facilitate meetings with all participants. This Zoom method was used to efficiently manage shift differences between participants. 

Meanwhile, the in-depth interviews were conducted face-to-face in a quiet and calm room using the semi-structured interview method while observing the participants’ attitudes and facial expressions. The FGD sessions were conducted twice, with each session lasting 73-82 minutes, and the in-depth interviews lasted 30-40 minutes. Data collection began on 4 September 2022 and was completed on 5 December 2022. The first data analysis was conducted for the first FGD, which was attended by 13 representative participants from four referral hospitals in a major city in a developing country, by ensuring the completeness of the transcripts and the researcher’s impression of the data quality. Anonymization was carried out during the analysis process by the experts of this study.

The second FGD was conducted to validate the results of the first FGD and to ensure that all participants had responded. It also provided an opportunity to ask any questions that had not been included in the guidelines during the first FGD. Data analysis was then conducted on the results of the in-depth interviews with the FGD participants who had worked in the NICU the longest. The interview process was recorded using a voice recorder. The main researcher ensured the completeness of the transcripts, documentation, daily notes and data quality. One member of the research team carried out the transcription and demographic data entry of all research data. All data were stored on Google Drive and were only accessible to the research team to ensure data security. Quotes were identified by analysing the results of the in-depth interviews with the experts in the research team. The researcher did not find any new coding in the second participant, so the researcher then took the third participant for confirmation. Thus, the total number of participants in the in-depth interviews was three out of 13 participants involved in the FGD activities. The inquiry audit method was used by the research team throughout the research process, including during the review of the findings. In addition, the researchers compared the observations, FGD results and in-depth interview results.

### Data analysis

 Microsoft Word was used to transcribe the results of the FGDs and interviews, and the themes were analyzed using qualitative content analysis ^( 15 )^ . The data analysis process began by entering the transcription results, field notes, and photographs showing nurses educating the mother of the infant into the ATLAS.ti software application version 8.4.16 to help the researchers interpret and develop concepts or hypotheses. The analysis was continued by looking at the pictures to find an empathetic look on the nurses’ face and reading the whole transcript 4-6 times to find significant statements that could describe the research content. The main researcher and family nurse coders marked each significant statement and identified the coding until no new coding was found. The main researcher then grouped the coding into categories, identified relationships between categories, identified themes and then developed concepts or hypotheses guided by two researchers as experts in paediatric nursing. The final step of the data analysis process was to verify the participants’ data through another Zoom meeting. In addition, the research team wrote up the study using the Consolidated Criteria for Reporting Qualitative Research (COREQ) checklist to describe the procedures used ^( 16 )^ . 

### Ethical considerations

Ethical approval was obtained from the Health Research Ethics Committee of each hospital (A, B, C, D) with nos. 1075/EC/KEPK-RSDK/2022, B/3233/070/10/2022, 420/10497 and 175 EC/KEPK/2021. All participants participated in this study without pressure or coercion. The informed consent form explained the aims, benefits and risks of the study. The researcher maintained confidentiality by using only the participant’s initials throughout the data analysis process, including in transcriptions and field notes. The code initials were P1 for the first participant, P2 for the second participant, and others are known only to the main researcher.

## Results

**Figure 1 -:**
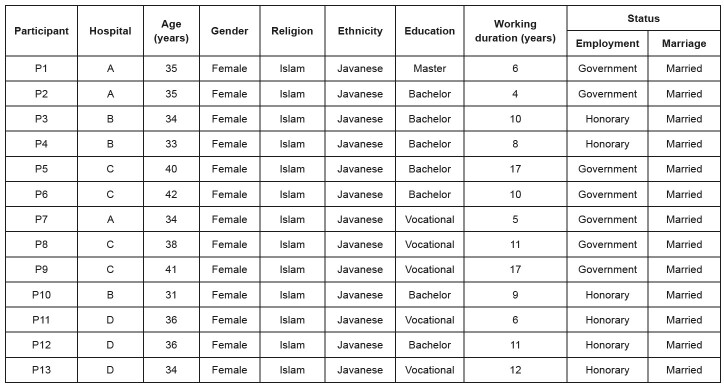
Participants’ characteristics. Semarang, Indonesia, 2022

 A total of 13 neonatal nurses met the inclusion criteria and participated in this study. The characteristics of the participants are shown in Figure 1 . 

Figure 1 shows that all participants were female, married, muslim and Javanese. As many as 46.2% of the participants had completed their bachelor’s degree in nursing and were government employees (53.8%). The mean age of the participants was 36.08 years, and the length of time they had worked in the NICU was 9.69 years. 

**Figure 2 -:**
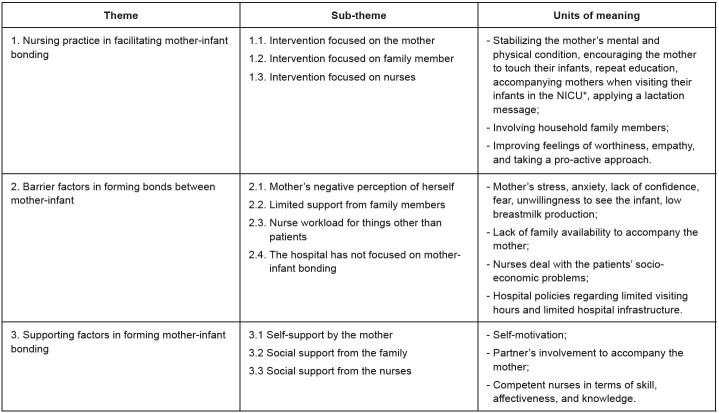
Themes, sub-themes, and units of meaning. Semarang, Indonesia, 2022

 The researchers identified three themes and 10 sub-themes ( Figure 2 ). These themes were identified after analysing the research data. 

 According to Figure 1 , the themes identified are explained as follows. 

Nursing practice in facilitating mother-infant bonding is described as what the nurses did with the mother, the family member, and the nurses themselves. In the mother-focused intervention, the participants played an active role in facilitating bonding between mothers and their premature babies. They improved the interaction between them by using an intervention that focused on stabilizing the mothers’ condition before they met their babies, encouraging them to physically touch their babies, providing repeated education, accompanying mothers when they visited their babies in the NICU, and providing lactation massages. These interventions are described in the following statements.

Participants expressed that stabilizing the mother’s condition is the first step in preparing her physically and psychologically to interact optimally with her baby. Here are some quotes:

*We need to stabilize their condition before we can teach them how to care for their premature babies, so that they can feel closer to their babies (P4).*

*The mothers have to be physically healthy first, we cannot help them bond if they are still physically ill. So we have to make sure they’re healthy before we can teach them about nutrition, mobilization, so they can interact with their baby (P8).*

*Psychological preparation is also important, as we said before, some mothers still feel fear and anxiety, to the point where they’re afraid to touch their baby, so we have to educate them in the beginning (P2).*

*NICU = Neonatal Intensive Care Unit

Participants also encouraged mothers who seemed reluctant to touch their babies. This was expressed in the following statements.

*We make it easier for mothers to touch their babies when they come to visit, even if they cannot breastfeed them directly.*

(P4)

*We need to build their confidence and reassure them that it’s OK to touch their baby and that a mother’s touch is very important for the baby.*

(P3)

Other participants mentioned that another common intervention was repeated training. Some of the statements made by the participants are as follows.

*We need to be more active or more frequent in educating mothers.*

(P11)

*We need to give education repeatedly, we need to build trust so that they remain confident and not afraid when they at least hold their baby.*

(P6)

The next intervention for the participants was to accompany the mother when she visited her baby in the NICU, as reported by the following participants.

*We have to accompany the mother during each visit, we have to build her confidence and convince her that it is okay to hold her baby and that a mother’s touch is very important for the baby...*

(P5)

*We observe and accompany them for a while until the mother and baby feel comfortable*

(P2).

Another intervention mentioned by the participants was breastfeeding massage when needed by preterm mothers. Below are some of the statements made by the participants:

*There have been cases where mothers have been given lactation massage and extra medication, but have not been able to produce breast milk*

(P1).

*For pain due to engorgement, we usually provide breast care by giving the mother a lactation massage to help her produce milk and relieve engorgement*

(P11).

Mother-centred care (sub-theme 1) illustrates that facilitating mother-infant bonding requires the mother’s willingness to accept preterm birth. In terms of family care, interventions that focused on family members involved other family members in caring for the preterm infant, as expressed by the following participants:

*As baby care follows the principle of family-centred care, other family members also need to be trained*

(P7).

*If the mother is tired or not well rested, another family member can take over and help. So we do not just train the father or mother, but also other family members who live in the same house as the patient*

(P13).

Involving family members, especially those living in the same house, is an alternative for some participants.

The third intervention focuses on the carer and improving their self-esteem, as reported by the following participants.

*The patient’s trust in me sometimes makes me feel valued*

(P5).

*It motivates us to accompany and support them, both with words and prayers*

(P13).

This valuable feeling motivates nurses to educate and motivate mothers to be close to their babies. It also evokes a feeling of empathy, which was expressed by the following participants.

*When we educate them, we try to put ourselves in the position of the mothers*

(P1).

*I would put myself in the mothers’ shoes*

(P7).

In addition, the nurses also tried to approach the mothers and be proactive, as expressed by the following participants.

*What we’ve done so far are approaches to convince the mother that nothing bad will happen as long as it’s done properly*

(P5).

*We have to approach them so that they remain confident and do not feel afraid, at least when they hold their baby*

(P9).

In the second theme, four sub-themes emerged that often evoke words/terms related to the environment for caring for preterm infants: mothers’ negative perceptions of themselves, limited support from family members, nurses’ workload for things other than patients, and hospitals that have not focused on facilitating mother-infant bonding.

Mothers with negative perceptions were a barrier for nurses in facilitating bonding. According to the participants, the barriers experienced by mothers, such as feeling stressed, anxious, fearful, lacking confidence, not wanting to see/touch their babies, lacking knowledge about caring for premature babies, and not producing breast milk, are also barriers for neonatal nurses in facilitating mother-baby bonding.

*The mother is stressed or anxious because she has not yet accepted how small her baby is*

(P1).

*Most mothers are afraid that they will feel tired, anxious and unsure. Even if it’s just to touch their baby or feed them*

(P2).

*Even to touch their baby, we still need to build the mothers’ confidence to do so*

(P11).

Another aspect of mothers’ negative perceptions expressed by the participants was the fear they feel when interacting with their preterm baby, as expressed by the following participants:

*The problem would be with the mother, especially if it’s her first child, she’s often afraid to hold her baby*

(P7).

*Usually for mothers of premature babies, they would feel scared and sad because it wasn’t time to have the baby and they see that their baby is very small*

(P8).

Participants also revealed that some mothers did not want to see their babies, especially in the case of primiparous mothers. The participants’ comments were as follows:

*Sometimes they don’t want to be close to their baby at all, or are too scared to visit, especially if the baby is attached to a lot of equipment*

(P3).

*Sometimes they do not even want to look at their baby*

(P5).

The mother’s lack of understanding about health was also mentioned as something that could become an obstacle for nurses in facilitating mother-infant bonding, as it had an impact on the mother’s minimal presence in the infant care room, as expressed by the following participants.

*Sometimes the problem is that the mother has little understanding of health and has parents who also lack this knowledge*

(P12)

*They may have a strong sense of anxiety or fear or lack of experience, which is common for most new mothers*

(P9).

Another aspect of mothers’ negative perceptions is their fear of not producing enough milk, which reduces their confidence in learning how to breastfeed optimally. Ultimately, this problem would affect the bonding between mother and baby. The following are examples of what the participants said:

*Breast milk that should be produced, but is not, will automatically cause the mother to regress and not come back to the hospital*

(P6).

*Sometimes the problem with breastfeeding is that mothers sometimes think too much about it, which makes it harder to produce breast milk*

(P7).

The next factor hindering mother-child bonding is the limited availability of family members to help mothers, as expressed by the following participants:

*There is another baby at home who needs care, so we can’t go to the hospital*

(P13).

*The problem we often face with low birth weight babies is that because their homes are far from the hospital, the mothers need help with transport, they cannot come to the hospital themselves, so they rely on their husbands to send them, and their husbands are not always available*

(P8).

The feeling of stress or anxiety felt by the mother, apart from the need to provide the best care and service for her baby, is a challenge for neonatal nurses when caring for patients. The challenge is even greater when nurses also have to deal with patients’ socio-economic problems and other responsibilities, as expressed by the following participants:

*For example, we cannot monitor them for a full 2 hours because we have other work to do*

(P3).

*Sometimes we have to solve problems that are not related to the patient’s condition, such as family problems. This happens a lot*

(P10).

*From the nurses’ point of view, we would take care of infants weighing as little as 900g for quite a long time, 100 days of care would cost 100 million rupiah in 2009/2010. At that time there was a mother who had a baby out of wedlock and without the blessing of her parents, this case required a lot of help from us nurses in terms of care and psychological help*

(P5).

Furthermore, prolonged engagement, where researchers observe nurses’ daily routines, often reveals communication problems between nurses and family members, leading to protests about the treatment provided. In addition, establishing a mother-child bond requires the involvement of multiple parties, including the hospital. Many participants reported that limited visiting hours and incomplete hospital infrastructure had a negative impact on mothers waiting for their babies to arrive at the hospital.

Most mothers do not want to wait in the waiting room because there is no bed associated with the NICU, and they usually feel uncomfortable squatting after giving birth, so they prefer to rest at home... Waiting rooms are important because they allow mothers to accompany their babies (P4).

We limit visiting hours in the NICU. In the NICU level 1 they can visit every 3 hours to breastfeed their baby, but for the NICU we still limit visiting hours to the morning and afternoon (P13).

Supporting factors for mothers of preterm babies are also provided by the participants to help them achieve their breastfeeding goals. These supporting factors consist of internal and external factors of mothers with a preterm baby. The internal factors are the mothers’ enthusiasm to be actively involved in caring for their baby, while the external factors are the husband’s involvement in supporting the mothers, as well as support from the participants themselves in relation to their competence as nurses. This was expressed by the following participants:

If the mother is well motivated, we are happy. Because if the mother is happy, the baby will be happy... (P11).

Thank God, in one case of a low birth weight baby, the father was able to drive his wife to the hospital for Kangaroo Mother Care (KMC). They usually drive their wives to the hospital before work and pick them up after work, that was our experience in the high-risk perinatal unit. So the baby can get breastmilk, because KMC also allows the baby to get breastmilk, and because the mother is motivated, it also helps the baby (P1).

If the mother produces little breast milk, we teach her how to express more (P12).

If the baby does not need oxygen, if it can feed perorally and its crying is strong, then we can teach it to drink and use the kangaroo method (P7).

We need to be able to motivate them (P9).

All the participants said that mothers need to have the skills to apply one of the principles of high-risk neonatal care, FCC, so that they can get used to caring for preterm babies at home. The use of FCC enables mothers to be actively involved in the care of their preterm babies. According to the nurses’ statement when the researcher did member checking with all the participants, the nurses said that they need some skills related to FCC, including lactation management, KMC, and special skills to improve bonding between mother and infant. Not all nurses in the four hospitals have received training in lactation management, but almost all have received training in KMC. However, it remains difficult to implement KMC in the absence of family members. KMC is usually done next to the baby incubator, and mothers do it more often because there are no visiting hours for fathers. Only one hospital had visiting hours for fathers, and then only for 15 minutes a day.

**Figure 3 -:**
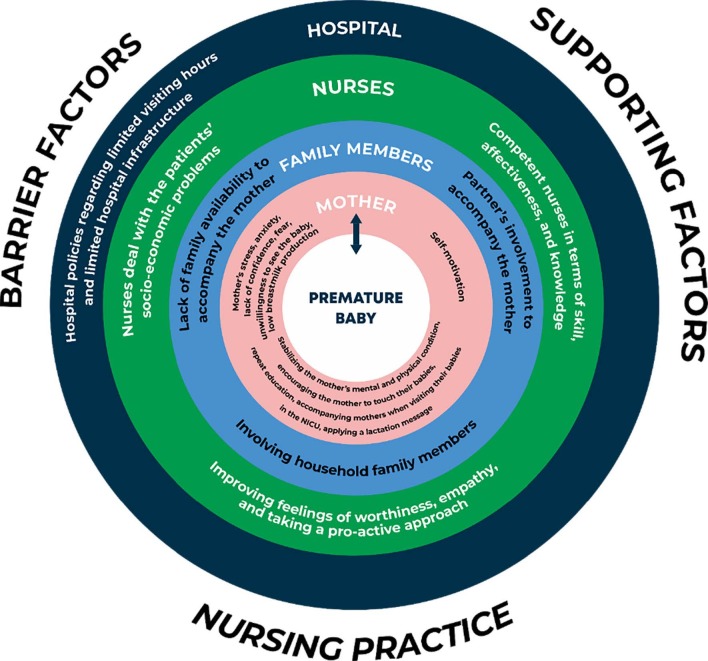
Nurses’ perceptions of facilitating bonding between mothers and premature babies in the neonatal unit

 The themes and sub-themes are integrated in Figure 3 . The figure shows that there are many factors that influence bonding between mothers and their babies. In the first layer it was premature babies with the white colour describing the sacredness. In the second layer it was the mothers with the pink colour describing mother to baby compassion. In the third layer, it was family members with the light blue colour describing strength for mother and premature babies. In the fourth layer, it was nurses with the green colour that describes wellness, it means that nurses are the person who helps to improve the level of infants, mother and their family. In the last layer, it was the hospital with the dark blue colour that describes sorrow on the mother’s perception. 

## Discussion

 The first theme shows us in detail what nurses did to facilitate bonding in the NICU. In the first sub-theme, the nurse focuses on caring for the infant’s mother. Through peer debriefing with the neonatal expert and the mental health expert, we interpret that stabilizing the infant’s mother is a crucial action by nurses at the first step in facilitating bonding. The second sub-theme also showed that family care interventions aimed at facilitating mother-infant bonding can be delivered through infant reassurance processes, which include providing comfort, touch and communication while the infant is in the incubator ^( 17 )^ . Involving family members also improves communication between the health care team and the family to reduce anxiety experienced by the family while waiting for the baby in the NICU ^( 18 )^ . In the third sub-theme, nurses described that they were motivated. The motivation was useful to face different challenges because at least three perspectives of Indonesian nurses in preparing discharge planning: internal barriers, external barriers and diversity in discharge planning ^( 19 )^ . The nurses faced these challenges with an empathetic attitude - they tried to put themselves in the mothers’ position, especially when providing education. 

 In this study the barrier factors were completed by mother, family member, nurse and hospital. To verify this result, an inquiry audit was carried out between the research team. Based on our beliefs, hospital infrastructure was the main problem that needed modification by hospital health policy to support nurses in facilitating bonding ^( 2^ , ^20 - 21 )^ . Some participants expressed that there is no bed for a family member while waiting for their baby. In addition, there are no visiting hours for fathers in the NICU and the socio-economic problems often faced by nurses are related to hospital infrastructure, especially the distance of the hospital from the mother’s home. The use of webcams could be the solution ^( 22 )^ but still unknown in developing countries, needed to verify its effectiveness when applied in developing countries. 

 Supporting factors included in this study consist of mother, family and nurses. In our view, the nurse’s competence can be an important factor in influencing the mother and the family member. Nurses need to teach bonding and infant interaction ^( 10 )^ . To improve parental sensitivity ^( 23 )^ , to help the mother or other family member to have skin-to-skin contact with the baby ^( 24^ - ^25 )^ , to provide effective communication ^( 8^ , ^26 )^ , to provide supportive counselling ^( 27 )^ , to improve nursing care, for example through music therapy ^( 28 )^ , and to ensure that interventions are applicable according to the hospital culture ^( 29 )^ . As nurses expressed that there is a workload, if the intervention is not applicable it will put a burden on the nurses themselves. 

 The three themes that emerged from this study could answer the overall aim of this research, which was to gain an in-depth understanding of nurses’ experiences in facilitating bonding between mothers and preterm babies. Nurses’ positive response when accompanying mothers with preterm babies can be a strength for nurses to provide the best care for mothers and their babies. Nurses need to control their emotions when they are with mothers and their families. Sympathy can interfere with the success of the nursing process. In addition, training for nurses is important to reduce work stress in level II NICUs and to increase work professionalism and empathy ^( 30 )^ . 

 It is important for a neonatal nurse to develop empathy as part of their role is to bridge the gap between mother and baby. Therefore, neonatal nurses need to understand the feelings and emotions that may be experienced by mothers with preterm babies ^( 4 )^ . All participants in this study agreed that nursing interventions cannot focus only on the infant, let alone on mother-infant bonding. Nursing interventions need to be comprehensive and address the entire external environment that creates mother-infant bonding, including the nurse who plays an important role in facilitating mother-infant interaction. 

 Neonatal nurses’ efforts to address these barriers are important in the NICU ^( 31 )^ . Listening, trusting and sharing knowledge are good efforts to make during mentoring. A successful relationship between neonatal nurses and mothers can be achieved through knowledge sharing, competence development and negotiation. In addition, the involvement of the father as a support system for the mother is also an important factor that can help nurses to overcome various barriers in supporting mothers of preterm babies. Studies also show that nurses are very positive about the involvement of fathers ^( 32 )^ . 

 Fathers play an important role throughout the life of their premature baby. Fathers have a major direct and indirect role in supporting the development of their NICU baby. Fathers can directly improve their baby’s development and health by providing kangaroo care, tactile stimulation, auditory stimulation and olfactory stimulation ^( 25 )^ . Then the indirect ways of supporting their babies were done by fathers through the coparenting alliance and providing financial support to the family. In terms of indirect ways, it can be said that the father indirectly influences the child’s development through the couple relationship ^( 33 )^ . This suggests that fathers need to be involved in programmes that support mother-child interactions. 

 This study suggests that one factor that supports mother-infant bonding is the infant’s immediate environment, which consists of the mother, caregivers and family members. Parental empowerment is the right step for parents to feel able to develop their roles as mothers or fathers, where one of the parental empowerments is emotional support ^( 34 )^ . Parents will receive this emotional support effectively if a nurse can support the mother by building her competence in caring skills, providing information and empathy, which can be formed from the nurse’s spiritual practices. 

 Spirituality is a domain characteristic that allows people to find purpose and meaning in life ^( 35 )^ . Parents usually find spiritual meaning in their own lives by focusing on the lives of their children. Therefore, receiving a premature baby in the NICU can be a traumatic experience for parents. Nurses who can recognize the existence of this spiritual meaning can empathize with and facilitate parents’ interaction with their infants and excel in their parental role, because empathy is a form of compassion, which is another related characteristic associated with well-developed personal spirituality ^( 36 )^ . This study has the limitation of not considering a health system from a specific geographical and cultural perspective. In this study, our participants were female and Muslim. We did not have male participants or participants from other religious backgrounds. Therefore, the results of this study may not reflect the nursing practice in male nurses’ views and especially in the intervention focused on the mother may not reflect the intervention ways in other religious. 

## Conclusion

From the perspective of the nurses in this study, efforts to facilitate mother-infant bonding can be well implemented if nurses have sufficient skills and are supported by adequate hospital infrastructure to make mothers comfortable while waiting for their preterm babies. Another important aspect is the support of family members, husbands and people around the mother to motivate her and increase her confidence. The results of this study can form the basis for a compilation of interventions that can be implemented in neonatal units, focusing on bonding efforts between mothers and their premature babies. The results of this study suggest the need for clear and well-organised standard operating procedures in the hospital regarding nursing intervention programmes to facilitate mother-baby bonding, starting when the mother enters the neonatal unit or even before. According to our study, it is important to first stabilise the mother’s mental health in order to facilitate bonding. Therefore, neonatal nurses need to liaise with other nurses in another unit, such as the labour ward or the obstetric polyclinic, to ensure that the mother’s mental health is good if she is likely to give birth to a premature baby.
