# Supplementary material for: Enhancing early bonding between mothers and preterm infants: findings from a Multicentre Qualitative Study of Nurses
Source: Rev Lat Am Enfermagem. 2024 Sep 23;32:e4200. doi: 10.1590/1518-8345.7034.4200 (PMC11421526; doi:10.1590/1518-8345.7034.4200)
Supplement: Translation

# Translation: Experiencias de enfermeros en la facilitación del vínculo precoz entre madres y bebés prematuros: un estudio cualitativo multicêntrico*

**DOI:** 10.1590/1518-8345.7034.4199

**Published:** 2024-09-23

**Authors:** Nopi Nur Khasanah, Yeni Rustina, Dessie Wanda, Iskim Luthfa

**Affiliations:** 1 Sultan Agung Islamic University, College of Nursing, Semarang, Indonesia, Asia; 2 University of Indonesia, College of Nursing, Depok, Indonesia, Asia; 3 Bolsista da Lembaga Pengelola Dana Pendidikan (LPDP), Indonesia

**Keywords:** Relaciones Familiares, Hospitales Urbanos, Bienestar del Lactante, Madres, Relaciones Madre-Hijo, Enfermeras Neonatales

## Abstract

**Objetivo:**

este estudio tiene como objetivo explorar las experiencias de los enfermeros neonatales en la facilitación del vínculo precoz entre madres y bebés prematuros.

**Método:**

se adoptó un enfoque cualitativo descriptivo, utilizando discusiones de grupos focales con 13 participantes de cuatro hospitales de referencia en una gran ciudad de un país en desarrollo. Posteriormente, se realizaron entrevistas en profundidad con tres participantes.

**Resultados:**

emergieron tres temas principales, destacando las barreras y los facilitadores para la creación de vínculos precoces entre madres y bebés prematuros. El estudio también exploró las prácticas de cuidado que buscan facilitar el vínculo precoz en el entorno social de los bebés prematuros, involucrando a madres, familias, enfermeras y hospitales.

**Conclusiones:**

las barreras y los facilitadores identificados informan el desarrollo de estrategias de intervención para enfermeros neonatales con el fin de promover el vínculo precoz. El vínculo precoz ideal requiere habilidades mejoradas de gestión de enfermería, infraestructura hospitalaria adecuada y la participación de la familia del paciente. Estos resultados contribuyen al avance del conocimiento sobre salud y enfermería.

## Destacados

**(1)** El vínculo precoz puede alcanzarse de manera ideal si cuenta con el apoyo de enfermeros, hospital y familia.

**(2)** Priorizar la estabilización de la condición de la madre antes de promover el vínculo madre-bebé.

**(3)** Las enfermeras neonatales juegan un papel fundamental en la promoción del vínculo precoz entre madre y bebé.

**(4)** Las intervenciones integrales deben adaptarse a las normas culturales del hospital.

**(5)** La participación de los padres puede ayudar a los enfermeros a superar varias barreras en el cuidado materno.

## Introducción

 La introducción del cuidado en unidades neonatales marca un cambio significativo en la práctica de enfermería, enfatizando la provisión de estímulos adecuados para el desarrollo de bebés prematuros ^( 1 )^ . Aunque los profesionales de enfermería pueden proporcionar estímulos, lo ideal es que los bebés prematuros los reciban directamente de sus madres. Además de promover el crecimiento y desarrollo ideales, la estimulación materna fortalece el vínculo entre la madre y el bebé prematuro. Desafortunadamente, los enfermeros neonatales a menudo ignoran el trabajo con los padres y se centran solo en las necesidades médicas del bebé, limitando así la participación de los padres y dejando de promover el vínculo entre las madres y los bebés prematuros ^( 2 )^ . Sin embargo, los enfermeros neonatales tienen una oportunidad valiosa de facilitar el establecimiento de lazos ^( 3 )^ entre las madres y sus bebés prematuros ^( 4 )^ . Investigaciones sugieren que el vínculo precoz entre la madre y el bebé prematuro tiene efectos positivos mientras el bebé aún está en la Unidad de Cuidados Intensivos Neonatales (UCIN) ^( 5 )^ . 

 Los enfermeros neonatales necesitan tener una comprensión diferenciada de las diversas estrategias para promover el vínculo entre las madres y sus bebés prematuros. Este entendimiento es esencial para la implementación del enfoque de Cuidados Centrados en la Familia (CCF) para los cuidados neonatales. Los enfermeros tienen un papel fundamental que desempeñar en la promoción de esta interacción, ya que el nacimiento prematuro puede desafiar la percepción de la mujer sobre la maternidad y hacer que se sienta inadecuada para cuidar o proteger al bebé. Tales sentimientos pueden impedir el desarrollo de un vínculo fuerte entre las madres y los bebés prematuros ^( 6 )^ . Además, las iniciativas para involucrar a los padres en la UCIN deben ser culturalmente sensibles para reducir las complicaciones del vínculo. Las necesidades de cada madre varían según sus circunstancias individuales ^( 7 )^ . Aquellas cuyos bebés necesitan cuidados en la UCIN generalmente requieren apoyo emocional de los profesionales de salud, además de información clara y comprensiva sobre los cuidados médicos de sus bebés, oportunidades para participar en la toma de decisiones e involucrarse en las actividades de cuidado del bebé ^( 8 )^ . 

 Las investigaciones sobre el papel de los enfermeros en el ambiente de la UCIN aún son limitadas, incluyendo investigaciones sobre la facilitación del vínculo precoz entre madres y bebés prematuros. Estudios de evaluación de necesidades de UCINs ^( 9 )^ identificaron que los enfermeros neonatales juegan un papel relevante tanto en la atención a las necesidades de los padres, cuando sus bebés están en tratamiento intensivo, como en relación con la salud de los bebés prematuros. La UCIN es un entorno terapéutico diseñado para cuidar de recién nacidos de alto riesgo. La condición inestable del bebé, los procedimientos riesgosos y el hostigamiento verbal de los padres son preocupaciones enfrentadas por los enfermeros de la UCIN ^( 10 )^ . Este estúdio, por lo tanto, tiene como objetivo explorar las experiencias de los enfermeros en la facilitación del vínculo entre las madres y sus bebés prematuros en la UCIN. 

## Método

### Diseño del estudio

 Este es un estudio descriptivo cualitativo con un enfoque interpretativo ^( 11 )^ . Dicho enfoque interpretativo puede explorar y describir problemas de investigación y resumir un fenómeno de manera integral ^( 12 )^ . Además, el equipo de investigación tiene un interés común en la salud y bienestar de bebés prematuros y está compuesto por tres mujeres especialistas en Enfermería Pediátrica y un hombre especialista en Enfermería Familiar. El equipo de investigación no garantiza una relación especial con ninguno de los participantes de este estudio. 

### Lugar y participantes

 Fue utilizado la técnica de discusión de grupo focal (GF), seguida de entrevistas en profundidad para corroborar los resultados. Los datos fueron recolectados mediante la aplicación *Zoom* , usando la técnica de GF y sesiones de entrevistas presenciales. Las actividades de GF se llevaron a cabo con el investigador principal y la enfermera de la familia. Además, la reunión del GF ocurrió dos veces. 

 La primera reunión se realizó por la tarde porque su naturaleza en línea permitió la participación de diferentes profesionales que trabajaban en distintos turnos (mañana, tarde y noche). De común acuerdo, la segunda reunión también se realizó por la tarde, ya que la mayoría de los enfermeros líderes de equipo trabajan en el turno de la mañana. Todos los enfermeros participaron en la primera reunión de GF en el mismo día y horario, usando la aplicación *Zoom* , solo dos enfermeros no participaron en la segunda reunión. Aunque hubo problemas de conexión con la red durante las actividades en línea del GF, los participantes respondieron a todas las preguntas. 

Las entrevistas en profundidad fueron conducidas solo por el investigador principal. Las entrevistas se realizaron cara a cara en la sala de entrenamiento de la unidad neonatal, inmediatamente después del turno de la mañana. La codificación fue realizada por el investigador principal y los resultados de la codificación fueron analizados por especialistas en enfermería pediátrica. La población de este estudio estuvo compuesta por enfermeros que trabajaron en la UCIN de septiembre a diciembre de 2022.

 Los participantes fueron seleccionados intencionalmente usando muestreo estratégico de casos críticos ^( 13 )^ para maximizar la información necesaria. Los investigadores explicaron los motivos de la investigación y organizaron las rutinas de los participantes por 2 a 3 horas/día durante tres meses para crear familiaridad con cuatro hospitales de referencia en una gran ciudad de un país en desarrollo. Los hospitales fueron seleccionados según los siguientes criterios: (1) hospitales públicos con una unidad neonatal, (2) hospital acreditado nacionalmente y (3) la unidad neonatal estaba implementando el CCF. Además, los participantes debían cumplir con los criterios de inclusión, que eran: haber trabajado en la UCIN por al menos cinco años, ser líder de equipo y poder comunicarse en línea. Solo había dos o tres líderes de equipo en cada UCIN del hospital. No hubo deserciones, ya que todos los participantes fueron cooperativos durante el proceso de recolección de datos. 

### Recolección de datos

 El instrumento utilizado en este estudio fue una guía general de preguntas relacionadas con las experiencias de los enfermeros en la facilitación del vínculo entre las madres y sus bebés prematuros. Las preguntas fueron desarrolladas de acuerdo con las respuestas de los participantes durante el GF y las entrevistas en profundidad. Se movilizaron varias herramientas de apoyo durante la recolección de datos, incluyendo papel de carta y hojas de notas de campo para registrar detalles importantes, como las expresiones faciales de los participantes y las actitudes hacia los investigadores durante la recolección de datos. En el estudio, se usó una grabadora de voz durante las entrevistas en profundidad con tres participantes. Sin embargo, el uso de la grabadora no fue necesario durante las actividades de GF, ya que estas se grabaron utilizando la aplicación de reunión *Zoom* . Los investigadores probaron las herramientas de recolección de datos antes de iniciar el proceso, mediante Grupos Focales (GFs) con cuatro enfermeros gestores de las salas de la UCIN en cada hospital donde se realizó el estudio, además de realizar entrevistas en profundidad con dos enfermeros. Los resultados de los GFs y las entrevistas fueron evaluados por expertos en enfermería pediátrica para obtener retroalimentación. 

 La primera etapa del proceso de recolección de datos fue identificar a los posibles participantes basándose en los criterios de inclusión definidos por el investigador principal. El gerente de la UCIN colaboró con el investigador principal informando cuáles enfermeros cumplían con los criterios y eran participantes potenciales. El investigador principal realizó una sesión de orientación con los posibles participantes para crear confianza entre ellos ^( 14 )^ . Todos los posibles participantes mostraron su disposición para firmar el consentimiento informado después de que el investigador principal explicó las metas, objetivos y procedimientos a seguir durante la investigación. La recolección de datos usando la técnica GF se realizó en línea en una sala *Zoom* para facilitar las reuniones con todos los participantes. El método *Zoom* fue utilizado para gestionar eficientemente las diferencias de turno entre los participantes. 

Las entrevistas en profundidad se realizaron cara a cara en una sala tranquila y silenciosa, utilizando entrevista semiestructurada mientras se observaban las actitudes y expresiones faciales de los participantes. Las sesiones de GF se realizaron dos veces, con cada sesión durando entre 73 y 82 minutos, mientras que las entrevistas en profundidad duraron entre 30 y 40 minutos. La recolección de datos comenzó el 4 de septiembre de 2022 y concluyó el 5 de diciembre de 2022. El primer análisis se realizó para el primer GF, que contó con la presencia de 13 participantes representando a cuatro hospitales de referencia en una gran ciudad, garantizando la integridad de las transcripciones y la impresión del investigador sobre la calidad de los datos. Se implementó el anonimato durante el proceso de análisis por los investigadores.

El segundo GF se realizó para validar los resultados del primero y para asegurar que todos los participantes hubieran respondido. El segundo GF, también proporcionó una oportunidad de hacer preguntas que no habían sido incluidas durante el primer GF. El análisis de los datos se realizó entonces basándose en los resultados de las entrevistas en profundidad realizadas con los participantes del GF que trabajaban en la UCIN desde hace más tiempo. El proceso de entrevista fue registrado con un grabador de voz.

 El investigador principal aseguró la integridad de las transcripciones, la documentación, las anotaciones diarias y la calidad de los datos. Un miembro del equipo de investigación realizó la transcripción y la entrada de todos os datos demográficos de la investigación. Todos los datos fueron almacenados en *Google Drive* y solo pudieron ser accedidos por el equipo de investigación para garantizar la seguridad de los datos. Las citas fueron identificadas mediante el análisis de los resultados de las entrevistas en profundidad con los expertos del equipo de investigación. 

En el proceso de codificación, cuando el investigador no encontró ninguna codificación nueva en la segunda entrevista, pasó a la tercera para confirmarla. Así, el número total de participantes en las entrevistas en profundidad fue tres, de los 13 participantes involucrados en las actividades de GF. El método de auditoría de investigación fue utilizado por el equipo de investigación durante todo el proceso de investigación, incluyendo durante la revisión de los resultados. Además, los investigadores compararon las observaciones, los resultados de los GF y los de las entrevistas en profundidad.

### Análisis de los datos

 El *Microsoft Word* fue utilizado para transcribir los resultados de los GF y las entrevistas, y los resultados fueron analizados mediante el análisis cualitativo de contenido ^( 15 )^ . De esta manera, el proceso de análisis de datos comenzó con la inserción de los resultados de la transcripción, las notas de campo y las fotografias, que mostraban a las enfermeras educando a las madres de los bebés, en el *software* ATLAS.ti, versión 8.4.16, para luego ayudar a los investigadores a interpretar y desarrollar conceptos o hipótesis. 

El análisis continuó con las observaciones de las fotos para encontrar una mirada empática en el rostro de las enfermeras y con las lecturas de la transcripción completa, realizadas de cuatro a seis veces para encontrar declaraciones significativas que pudieran describir el contenido de la investigación.

 El investigador principal y los codificadores especialistas en enfermería familiar marcaron cada declaración significativa e identificaron los códigos hasta que no se encontrara ninguna nuevo código. Luego, el investigador principal agrupó los códigos en categorías, identificó las relaciones entre ellos y los temas y desarrolló conceptos o hipótesis guiados por dos investigadores especialistas en enfermería pediátrica. La etapa final del proceso de análisis de datos fue la verificación de la información de los participantes mediante otra reunión de *Zoom* . Además, el equipo de investigación escribió el estudio usando la lista de verificación *Consolidated Criteria for Reporting Qualitative Research* (COREQ) para describir los procedimientos utilizados ^( 16 )^ . 

### Consideraciones éticas

La aprobación ética fue obtenida por el Comité de Ética en Investigación en Salud de cada hospital (A, B, C, D) con los números 1075/EC/KEPK-RSDK/2022, B/3233/070/10/2022, 420/10497 y 175 EC/KEPK/2021. Todos los participantes colaboraron en este estudio sin presión o coacción. El formulario de consentimiento informado explicó los objetivos, beneficios y riesgos del estudio. El investigador mantuvo la confidencialidad utilizando solo las iniciales “P” seguido de la numeración para los participantes durante todo el proceso de análisis de datos, incluidas las transcripciones y las notas de campo. Las iniciales de código fueron P1 para el primer participante, P2 para el segundo participante, así sucesivamente.

## Resultados

 Un total de 13 enfermeras neonatales cumplió con los criterios de inclusión y participó en este estudio. Las características de los participantes están presentadas en la Figura 1 . 

**Figura 1 -:** Características de los participantes. Semarang, Indonesia, 2022

 La Figura 1 muestra que todos los participantes eran mujeres, casadas, musulmanas y javanesas; 46,2% había completado el bachillerato en enfermería y eran funcionarias públicas (53,8%). La edad promedio de las participantes era de 36,08 años y el tiempo de trabajo en la UCIN de 9,69 años. 

 Los investigadores establecieron tres temas, 10 subtemas y sus unidades de significado ( Figura 2 ), según los resultados de la investigación. 

 Conforme la Figura 2 , la facilitación del vínculo madre-bebé se describe cómo las prácticas de enfermería realizadas con la madre, con los familiares y con las propias enfermeras. En la intervención enfocada en la madre, las participantes desempeñaron un papel activo en el proceso de facilitación. La interacción se mejoró utilizando una intervención que se centró en estabilizar la condición de las madres antes de que conocieran a sus bebés, animándolas a tocar físicamente a sus bebés, proporcionando educación repetida, acompañándolas cuando visitaban a sus bebés en la UCIN y proporcionando masajes de lactancia. Tales intervenciones se describen en los relatos a continuación. 

**Figura 2 -:** Temas, subtemas y unidades de significado. Semarang, Indonesia, 2022

*UCIN = Unidad de Cuidados Intensivos Neonatal

Las participantes expresaron que la estabilización de la condición de la madre es el primer paso para prepararla física y psicológicamente para interactuar de manera óptima con el bebé. A continuación, algunos de los extractos que indican tales aspectos:

*Tenemos que estabilizar su condición antes de enseñarles a cuidar a sus bebés prematuros, para que puedan sentirse más cercanas a sus bebés.*

(P4)

*Las madres necesitan estar físicamente saludables primero, no podemos ayudarlas a crear vínculos si todavía están físicamente enfermas. Por lo tanto, tenemos que asegurarnos de que estén saludables antes de enseñarles sobre nutrición, movilización, para que puedan interactuar con el bebé.*

(P8)

*La preparación psicológica también es importante, pues, como dijimos antes, algunas madres aún sienten miedo y ansiedad, hasta el punto de tener miedo de tocar al bebé, por eso tenemos que educarlas desde el principio.*

(P2)

Las participantes también alentaron a las madres que parecían reticentes a tocar a sus bebés, como se puede observar a continuación:

*Facilitamos que las madres toquen a sus bebés cuando vienen a visitarnos, incluso si no pueden amamantarlos directamente.*

(P4)

*Necesitamos aumentar su confianza y asegurarles que no hay problema en tocar al bebé y que el contacto de la madre es muy importante para el bebé.*

(P3)

Otras participantes mencionaron que otra intervención común era la educación repetida, como ejemplifican los siguientes extractos:

*Necesitamos ser más activos o más frecuentes en la educación de las madres.*

(P11)

*Necesitamos dar educación repetidamente, necesitamos crear confianza para que ellas permanezcan confiadas y no tengan miedo cuando al menos sostengan al bebé.*

(P6)

La siguiente intervención de las participantes fue acompañar a la madre cuando visitaba al bebé en la UCIN, según lo relatado:

*Debemos acompañar a la madre durante cada visita, debemos aumentar su confianza y convencerla de que no hay problema en sostener al bebé y que el contacto de la madre es muy importante para el bebé...*

(P5)

*Observamos y acompañamos por un tiempo hasta que la madre y el bebé se sientan cómodos.*

(P2)

Otra intervención mencionada por las participantes fue el masaje para la lactancia, cuando era necesario. A continuación, algunos de los relatos:

*Hubo casos en que las madres recibieron masaje de lactancia y medicación adicional, pero no lograron producir leche materna.*

(P1)

*Para el dolor causado por el ingurgitamiento, generalmente proporcionamos cuidados con el pecho haciendo un masaje de lactancia para ayudar a la madre a producir leche y aliviar el ingurgitamiento.*

(P11)

El cuidado centrado en la madre (subtema 1) ilustra que la facilitación del vínculo madre-bebé requiere la disposición de la madre para aceptar el nacimiento prematuro. En cuanto a los cuidados con la familia, las intervenciones que se centraron en los miembros de la familia implicaron a otros miembros de la familia en el cuidado del bebé prematuro, según se informa:

*Como el cuidado del bebé sigue el principio del cuidado centrado en la familia, otros miembros de la familia también necesitan ser entrenados.*

(P7)

*Si la madre está cansada o no está bien descansada, otro miembro de la familia puede tomar el control y ayudar. Por lo tanto, no entrenamos solo al padre o a la madre, sino también a otros miembros de la familia que viven en la misma casa que el paciente.*

(P13)

Involucrar a los miembros de la familia, especialmente a aquellos que viven en la misma casa, es una alternativa para algunas participantes.

La tercera intervención se centra en el cuidador y en la mejora de su autoestima, según lo comentado por las siguientes participantes:

*La confianza del paciente en mí a veces me hace sentir valorado.*

(P5)

*Eso nos motiva a acompañarlos y apoyarlos, tanto con palabras como con oraciones.*

(P13)

El sentimiento valioso descrito motivó a las enfermeras a educar y a incentivar a las madres a estar cerca de sus bebés. Tal sentimiento también evocó la empatía, expresada por las participantes:

*Cuando los educamos, intentamos ponernos en la posición de las madres.*

(P1)

*Yo me pondría en el lugar de las madres.*

(P7)

Además, las enfermeras también intentaron acercarse a las madres y ser proactivas, según se informó:

*Lo que hemos hecho hasta ahora son enfoques para convencer a la madre de que nada malo sucederá, siempre que se haga correctamente.*

(P5)

*Tenemos que acercarnos a ellas para que permanezcan confiadas y no sientan miedo, al menos cuando sostienen al bebé.*

(P9)

En el segundo tema, surgieron cuatro subtemas que frecuentemente evocan palabras/términos relacionados con el ambiente hospitalario en el cuidado de bebés prematuros, a saber: percepciones negativas de las madres sobre sí mismas; apoyo limitado de los miembros de la familia; carga de trabajo de los enfermeros para otras actividades además de los cuidados a los pacientes y hospitales que no se concentran en facilitar el vínculo madre-bebé.

Las madres con percepciones negativas dificultan el desarrollo de prácticas por parte de las enfermeras para la facilitación del vínculo. Según las participantes, las barreras enfrentadas por las madres – como estrés, ansiedad, miedo, falta de confianza, no querer ver/tocar a sus bebés, falta de conocimiento sobre cómo cuidar a bebés prematuros y no producir leche materna – también son barreras para las enfermeras neonatales en facilitar el vínculo madre-bebé:

*La madre está estresada o ansiosa porque aún no ha aceptado el hecho de que el bebé sea pequeño.*

(P1)

*La mayoría de las madres tienen miedo de sentirse cansadas, ansiosas e inseguras. Incluso si es solo para tocar al bebé o alimentarlo.*

(P2)

*Incluso para tocar al bebé, todavía necesitamos aumentar la confianza de las madres para hacerlo.*

(P11)

Otro aspecto de las percepciones negativas de las madres expresado por las participantes fue el miedo que sienten al interactuar con el bebé prematuro, como se puede ver a continuación:

*El problema sería con la madre, especialmente si es su primer hijo, generalmente tiene miedo de sostener al bebé.*

(P7)

*Normalmente, las madres de bebés prematuros se sienten asustadas y tristes porque no era el momento de tener al bebé y ven que el bebé es muy pequeño.*

(P8)

Las participantes también revelaron que algunas madres no querían ver a sus bebés, especialmente en el caso de madres primerizas. Los comentarios de las participantes fueron los siguientes:

*A veces, no quieren estar cerca del bebé o tienen mucho miedo de visitarlo, especialmente si el bebé está conectado a muchos equipos.*

(P3)

*A veces, no quieren ni mirar al bebé.*

(P5)

La falta de comprensión de la madre sobre la salud también fue mencionada como algo que podría convertirse en un obstáculo para las enfermeras en la facilitación del vínculo madre-bebé, ya que esto tenía un impacto sobre la presencia mínima de la madre en la sala de cuidados con el bebé, como se informó:

*A veces, el problema es que la madre tiene poco conocimiento sobre salud y tiene padres que tampoco tienen ese conocimiento.*

(P12)

*Ellas pueden tener una fuerte sensación de ansiedad, miedo o falta de experiencia, lo que es común para la mayoría de las nuevas madres.*

(P9)

Además, otro aspecto de las percepciones negativas de las madres fue el miedo a no producir suficiente leche, lo que reduce su confianza en aprender a amamantar de forma ideal. A continuación, se pueden observar ejemplos de lo que las participantes reportaron:

*La leche materna que debería ser producida, pero no lo es, automáticamente hará que la madre retroceda y no vuelva al hospital.*

(P6)

*A veces, el problema con la lactancia es que las madres piensan demasiado en ello, lo que dificulta la producción de leche materna.*

(P7)

El próximo factor que dificultó el vínculo entre madre e hijo fue la disponibilidad limitada de miembros de la familia para ayudar a las madres, como se informó:

*Hay otro bebé en casa que necesita cuidados, por eso no podemos ir al hospital.*

*El problema que a menudo enfrentamos con bebés de bajo peso al nacer es que, como sus casas están lejos del hospital, las madres necesitan ayuda con el transporte, no pueden ir al hospital solas, entonces dependen de los esposos para enviarlas, y los esposos no siempre están disponibles (P8).*

(P13)

El sentimiento de estrés o ansiedad sentido por la madre, así como la necesidad de ofrecer el mejor cuidado y servicio para su bebé, fue un desafío para las enfermeras neonatales al cuidar a las pacientes. La situación es aún más desafiadora cuando las enfermeras también tienen que lidiar con los problemas socioeconómicos de las pacientes y otras responsabilidades, como se reportó:

*Por ejemplo, no podemos monitorearlas por dos horas enteras porque tenemos otro trabajo que hacer.*

(P3)

*A veces, tenemos que resolver problemas que no están relacionados con la condición del paciente, como problemas familiares. Eso sucede mucho.*

(P10)

*Desde el punto de vista de las enfermeras, cuidamos de bebés de apenas 900g durante un largo período, 100 días de cuidados costarían 100 millones de rupias en 2009/2010. En ese tiempo, había una madre que tuvo un bebé fuera del matrimonio y sin la bendición de los padres, y ese caso requirió mucha ayuda de nosotras, las enfermeras, en términos de cuidados y ayuda psicológica.*

(P5)

Además, la implicación prolongada, donde los investigadores observan las rutinas diarias de los enfermeros, a menudo revela problemas de comunicación entre los enfermeros y los miembros de la familia, lo que lleva a protestas sobre el tratamiento proporcionado. Además, el establecimiento de un vínculo entre madre e hijo requiere la implicación de varias partes, incluido el hospital. Muchos participantes mencionaron que el horario limitado de visitas y la infraestructura incompleta del hospital tuvieron un impacto negativo en las madres que esperaban la llegada de sus bebés al hospital.

*La mayoría de las madres no quiere esperar en la sala de espera porque no hay cama asociada a la UCIN, y generalmente se sienten incómodas agachadas después del parto, por lo que prefieren descansar en casa... Las salas de espera son importantes porque permiten que las madres acompañen a sus bebés.*

(P4)

*Limitamos el horario de visitas en la UCIN. En la UCIN nivel 1, pueden visitar cada 3 horas para amamantar al bebé, pero en la UCIN aún limitamos el horario de visita a la mañana y la tarde.*

(P13)

Los factores de apoyo para madres de bebés prematuros también son proporcionados por las participantes para ayudarlas a alcanzar sus metas de lactancia. Estos factores consisten en internos y externos; y los internos son el entusiasmo de las madres por involucrarse activamente en el cuidado del bebé, mientras que los externos son la implicación del esposo en apoyar a las madres, así como el apoyo de las propias participantes en relación a su competencia como enfermeras, como se reporta:

*Si la madre está bien motivada, nos sentimos felices. Porque si la madre está feliz, el bebé estará feliz...*

(P11)

*Gracias a Dios, en un caso de bebé con bajo peso al nacer, el padre pudo llevar a la esposa al hospital para el Método Madre Canguro (MMC). Generalmente llevan a sus esposas al hospital antes del trabajo y las recogen después del trabajo, esa fue nuestra experiencia en la unidad perinatal de alto riesgo. Así, el bebé puede recibir leche materna, porque el MMC también permite que el bebé reciba leche materna y, como la madre está motivada, esto también ayuda al bebé.*

(P1)

*Si la madre produce poca leche materna, le enseñamos a extraer más.*

(P12)

*Si el bebé no necesita oxígeno, si puede alimentarse por vía oral y su llanto es fuerte, podemos enseñarle a beber y usar el método canguro.*

(P7)

*Necesitamos ser capaces de motivarlas.*

(P9)

Todas las participantes dijeron que las madres necesitan tener las habilidades necesarias para aplicar uno de los principios del cuidado neonatal de alto riesgo, el CCF, para que puedan acostumbrarse a cuidar de bebés prematuros en casa. El uso del CCF permite que las madres se involucren activamente en el cuidado de sus bebés prematuros. Según la declaración de las enfermeras, cuando el investigador hizo la verificación de los miembros con todos las participantes, las enfermeras dijeron que necesitaban algunas habilidades relacionadas con el CCF, incluyendo el manejo de la lactancia, el Método Madre Canguro (MMC) y las habilidades especiales para mejorar el vínculo entre la madre y el bebé. No todas las enfermeras de los cuatro hospitales recibieron entrenamiento en manejo de la lactancia, pero casi todas recibieron entrenamiento en MMC. Sin embargo, sigue siendo difícil implementarlo en ausencia de miembros de la familia.

El MMC generalmente se hace al lado de la incubadora de bebés y las madres lo hacen con más frecuencia porque no hay horario de visita para los padres, solo un hospital tenía horario de visita para los padres, y solo por 15 minutos al día.

**Figura 3 -:** Percepciones de las enfermeras sobre la facilitación del vínculo entre madres y bebés prematuros en la unidad neonatal

 Los temas y subtemas están integrados en la Figura 3 , la cual muestra los muchos factores que influyen en el vínculo entre las madres y sus bebés. En la primera capa, se tienen los bebés prematuros, con el color blanco describiendo la sacralidad. En la segunda capa, se muestran las madres, con el color rosa describiendo la compasión entre madre y bebé. En la tercera capa estaban los miembros de la familia, con el color azul claro, describiendo la fuerza de la madre y de los bebés prematuros. En la cuarta capa, se presentan los enfermeros, con el color verde, que describe el bienestar, de modo que remite a los enfermeros como sujetos que contribuyen a la mejora de los cuadros de los bebés, de las madres y, consecuentemente, de las familias. En la última capa, el hospital aparece con el color azul oscuro, el cual describe la tristeza en la percepción de la madre. 

## Discusión

El primer tema nos muestra en detalle lo que hicieron las enfermeras para facilitar el vínculo en la UCIN. En el primer subtema, la enfermera se concentra en el cuidado de la madre del bebé. Mediante el debate entre pares con el especialista en neonatología y el especialista en salud mental, interpretamos que la estabilización de la madre del bebé es una acción crucial de los enfermeros en la primera etapa de la facilitación del vínculo.

 El segundo subtema también mostró que las intervenciones de asistencia a la familia con el objetivo de facilitar el vínculo madre-bebé pueden realizarse mediante procesos de tranquilización del bebé, lo que incluye proporcionar confort, tacto y comunicación mientras el bebé está en la incubadora ^( 17 )^ . La participación de los miembros de la familia también mejora la comunicación entre el equipo de salud y la familia, para así reducir la ansiedad vivida por los familiares mientras esperan al bebé en la UCIN ^( 18 )^ . 

 En el tercer subtema, las enfermeras describieron que estaban motivados. Tal factor fue útil para enfrentar diferentes desafíos, considerando que hay al menos tres perspectivas de los enfermeros indonesios en la preparación del plan de alta: barreras internas, barreras externas y diversidad en la planificación del alta ^( 19 )^ . Las enfermeras enfrentaron estos desafíos con una actitud empática e intentaron ponerse en la posición de las madres, principalmente en la educación. 

 En este estudio, los factores de barrera fueron identificados por la madre, el miembro de la familia, la enfermera y el hospital. Para verificar este resultado, se realizó una auditoría de investigación entre el equipo de investigación. Basado en nuestras creencias, la infraestructura hospitalaria fue el principal problema que necesitaba ser modificado por la política de salud del hospital para apoyar a los enfermeros en la facilitación del vínculo ^( 2^ , ^20 - 21 )^ . Algunas participantes expresaron que no hay cama para un miembro de la familia mientras espera al bebé. Además, no hay horario de visita para los padres en la UCIN y los problemas socioeconómicos a menudo enfrentados están relacionados con la infraestructura hospitalaria, especialmente la distancia del hospital respecto a la casa de la madre. El uso de *webcams* podría ser una solución ^( 22 )^ pero aún es desconocido en los países en desarrollo, siendo necesario verificar su eficacia cuando se aplica en países en ese mismo contexto. 

 Los factores de apoyo incluidos en este estudio son: la madre, la familia y las enfermeiras, y la competencia de la enfermera puede ser un factor importante para influenciar a la madre y al miembro de la familia. Los enfermeros necesitan enseñar a crear vínculos y a interactuar con el bebé ^( 10 )^ , mejorar la sensibilidad de los padres ^( 23 )^ , ayudar a la madre o a otro miembro de la familia a tener contacto piel con piel con el bebé ^( 24^ - ^25 )^ , proporcionar una comunicación efectiva ^( 8^ , ^26 )^ , ofrecer asesoramiento de apoyo ^( 27 )^ , mejorar los cuidados de enfermería, por ejemplo, a través de la musicoterapia ^( 28 )^ , así como asegurar que las intervenciones sean aplicables de acuerdo con la cultura del hospital ^( 29 )^ . Como las enfermeras reportaron que hay una carga de trabajo, si la intervención no es aplicable, esto sobrecargará a las propias enfermeras. 

 Los tres temas que emergieron de este estudio podrían responder al objetivo general de esta investigación, que era obtener una comprensión profunda de las experiencias de los enfermeros en la facilitación del vínculo entre madres y bebés prematuros. La respuesta positiva de los enfermeros al acompañar a madres con bebés prematuros puede ser un punto fuerte para que ofrezcan el mejor cuidado para las madres y sus bebés. Los enfermeros necesitan controlar sus emociones cuando están con las madres y sus familias. La afinidad puede interferir en el éxito del proceso de enfermería. Además, el entrenamiento para enfermeros es importante para reducir el estrés en el trabajo en UCINs de nivel II y para aumentar el profesionalismo y la empatía en el trabajo ^( 30 )^ . 

 Es importante que una enfermera neonatal desarrolle empatía, ya que parte de su función es hacer de puente entre la madre y el bebé. Por lo tanto, las enfermeras neonatales necesitan entender los sentimientos y emociones que pueden experimentar las madres de bebés prematuros ^( 4 )^ . Todas las participantes de este estudio coincidieron en que las intervenciones de enfermería no pueden centrarse solo en el bebé, mucho menos en el vínculo madre-bebé. Las intervenciones de enfermería deben ser integrales y abordar todo el entorno externo que crea el vínculo madre-bebé, incluido el profesional de enfermería, que juega un papel importante en la facilitación de la interacción madre-bebé. 

 Los esfuerzos de los enfermeros neonatales para enfrentar estas barreras son importantes en la UCIN ^( 31 )^ . Escuchar, confiar y compartir conocimiento son esfuerzos que se deben realizar durante la mentoría. Una relación exitosa entre enfermeras neonatales y madres puede lograrse mediante el intercambio de conocimientos, el desarrollo de competencias y la negociación. Además, la participación del padre como un sistema de apoyo para la madre también es un factor importante que puede ayudar a los enfermeros a superar varias barreras en el apoyo a madres de bebés prematuros. Los estudios también muestran que los enfermeros son muy positivos respecto al involucramiento de los padres ^( 32 )^ . 

 Los padres desempeñan un papel importante durante toda la vida del bebé prematuro y tienen un papel importante, directo e indirecto, en el apoyo al desarrollo del bebé en la UCIN. Los padres pueden mejorar directamente el desarrollo y la salud del bebé promoviendo el método canguro, estimulación táctil, auditiva y olfativa ^( 25 )^ . Las formas indirectas de apoyo a los bebés fueron realizadas por los padres mediante la alianza de coparentalidad y el apoyo financiero a la familia. En términos de formas indirectas, se puede decir que el padre influye indirectamente en el desarrollo del niño a por medio de la relación de pareja ^( 33 )^ . Esto sugiere que los padres necesitan ser incluidos en programas que apoyen las interacciones entre madre e hijo. 

 Este estudio sugiere que un factor que apoya el vínculo entre madre e hijo es el entorno inmediato del bebé, que consiste en la madre, los cuidadores y los miembros de la familia. “El empoderamiento de los padres es el paso correcto para que se sientan capaces de desarrollar sus funciones como madres o padres, siendo uno de los empoderamientos de los padres el apoyo emocional” ^( 34 )^ . Los padres recibirán ese apoyo emocional de manera efectiva si una enfermera puede apoyar a la madre, desarrollando su competencia en habilidades de cuidado, proporcionando información y empatía, que pueden ser formadas a partir de las prácticas espirituales de la enfermera. 

 La espiritualidad es una característica de dominio que permite que las personas encuentren propósito y significado en la vida ^( 35 )^ . Los padres generalmente encuentran significado espiritual en sus propias vidas al centrarse en la vida de sus hijos. Por lo tanto, recibir un bebé prematuro en la UCIN puede ser una experiencia traumática para los padres. Los enfermeros que logran reconocer la existencia de ese significado espiritual pueden sentir empatía y facilitar la interacción de los padres con sus bebés, de manera que se destaquen en su papel de padres porque la empatía es una forma de compasión, es decir, otra característica relacionada asociada a la espiritualidad personal bien desarrollada ^( 36 )^ . Este estudio tiene la limitación de no considerar un sistema de salud en una perspectiva geográfica y cultural específica. En este estudio, nuestros participantes eran mujeres y musulmanas. No tuvimos participantes hombres o de otros orígenes religiosos. Por lo tanto, los resultados de este estudio pueden no reflejar la práctica de enfermería desde la perspectiva de enfermeros hombres y, especialmente, la intervención enfocada en la madre puede no reflejar las formas de intervención en otras religiones. 

## Conclusión

Desde el punto de vista de las enfermeras de este estudio, los esfuerzos para facilitar el vínculo madre-bebé pueden implementarse bien si los enfermeros tienen habilidades suficientes y cuentan con el apoyo de una infraestructura hospitalaria adecuada para que las madres se sientan cómodas mientras esperan a sus bebés prematuros. Otro aspecto importante es el apoyo de miembros de la familia, de los esposos y de personas alrededor de la madre para motivarla y aumentar su confianza. Los resultados de este estudio pueden formar la base para una compilación de intervenciones que pueden implementarse en unidades neonatales, enfocándose en los esfuerzos de vinculación entre madres y sus bebés prematuros. Los resultados de este estudio también sugieren la necesidad de procedimientos operativos estándar y organizados en el hospital en relación con los programas de intervención de enfermería para facilitar el vínculo madre-bebé, comenzando cuando la madre entra en la unidad neonatal o incluso antes. Según nuestro estudio, es importante primero estabilizar la salud mental de la madre para facilitar el vínculo. Por lo tanto, los enfermeros neonatales necesitan ponerse en contacto con otros enfermeros de otra unidad, como la sala de partos o la policlínica obstétrica para asegurarse de que la madre esté bien, especialmente si hay riesgo de parto prematuro.

# Translation: Experiências de enfermeiros na facilitação do vínculo precoce entre mães e bebês prematuros: um estudo qualitativo multicêntrico *

**DOI:** 10.1590/1518-8345.7034.4201

**Published:** 2024-09-23

**Authors:** Nopi Nur Khasanah, Yeni Rustina, Dessie Wanda, Iskim Luthfa

**Affiliations:** 1 Sultan Agung Islamic University, College of Nursing, Semarang, Indonesia, Asia; 2 University of Indonesia, College of Nursing, Depok, Indonesia, Asia; 3 Bolsista da Lembaga Pengelola Dana Pendidikan (LPDP), Indonésia

**Keywords:** Relações Familiares, Hospitais Urbanos, Bem-Estar do Lactente, Mães, Relações Mãe-Filho, Enfermeiras Neonatologistas

## Abstract

**Objetivo:**

este estudo tem como objetivo explorar as experiências dos enfermeiros neonatais na facilitação do vínculo precoce entre mães e bebês prematuros

**Método:**

foi adotada uma abordagem qualitativa descritiva, usando discussões de grupos focais com 13 participantes de quatro hospitais de referência em uma grande cidade de um país em desenvolvimento. Em seguida, foram realizadas entrevistas em profundidade com três participantes

**Resultados:**

surgiram três temas principais, destacando as barreiras e os facilitadores para a criação de vínculos precoces entre mães e bebês prematuros. O estudo também explorou as práticas de cuidados que visam a facilitar o vínculo precoce no ambiente social dos bebês prematuros, envolvendo mães, famílias, enfermeiras e hospitais.

**Conclusões:**

as barreiras e os facilitadores identificados informam o desenvolvimento de estratégias de intervenção para enfermeiros neonatais a fim de promover o vínculo precoce. O vínculo precoce ideal requer habilidades aprimoradas de gerenciamento de enfermagem, infraestrutura hospitalar adequada e envolvimento da família do paciente. Esses resultados contribuem para o avanço do conhecimento sobre saúde e enfermagem.

## Destaques

**(1)** O vínculo precoce pode ser alcançado de forma ideal se for apoiado por enfermeiros, hospital, família.

**(2)** Priorizar a estabilização da condição da mãe antes de promover o vínculo mãe-bebê.

**(3)** As enfermeiras neonatais desempenham um papel fundamental na promoção do vínculo precoce entre mãe e bebê.

**(4)** Intervenções abrangentes devem ser adaptadas às normas culturais do hospital.

**(5)** O envolvimento dos pais pode ajudar os enfermeiros a superar várias barreiras ao cuidado materno.

## Introdução

 A introdução do cuidado em unidades neonatais marca uma mudança significativa na prática de enfermagem, enfatizando o fornecimento de estímulos adequados para o desenvolvimento de bebês prematuros ^( 1 )^ . Embora os profissionais de enfermagem possam fornecer estímulos, o ideal é que os bebês prematuros os recebam diretamente de suas mães. Além de promover o crescimento e o desenvolvimento ideais, a estimulação materna fortalece o vínculo entre a mãe e o bebê prematuro. Infelizmente, os enfermeiros neonatais muitas vezes ignoram o trabalho com os pais e se concentram apenas nas necessidades médicas do bebê, de forma a limitar a participação dos pais, deixando de promover o vínculo entre as mães e os bebês prematuros ^( 2 )^ . No entanto, os enfermeiros neonatais têm uma oportunidade valiosa de facilitar o estabelecimento de laços ^( 3 )^ entre as mães e seus bebês prematuros ^( 4 )^ . Pesquisas sugerem que o vínculo precoce entre a mãe e o bebê prematuro tem efeitos positivos enquanto o bebê ainda está na Unidade de Terapia Intensiva Neonatal (UTIN) ^( 5 )^ . 

 Os enfermeiros neonatais precisam ter uma compreensão diferenciada das diversas estratégias para promover o vínculo entre as mães e seus bebês prematuros. Esse entendimento é essencial para a implementação da abordagem de Cuidados Centrados na Família (CCF) para os cuidados neonatais. Os enfermeiros têm um papel fundamental a desempenhar na promoção dessa interação, pois o nascimento prematuro pode desafiar a percepção da mulher sobre a maternidade e fazer com que ela se sinta inadequada para cuidar ou proteger o bebê. Tais sentimentos podem impedir o desenvolvimento de um vínculo forte entre as mães e os bebês prematuros ^( 6 )^ . Além disso, as iniciativas para envolver os pais na UTIN devem ser culturalmente sensíveis para reduzir as complicações do vínculo. As necessidades de cada mãe variam de acordo com suas circunstâncias individuais ^( 7 )^ . Aquelas cujos bebês precisam de cuidados na UTIN geralmente demandam apoio emocional dos profissionais de saúde, além de informações claras e abrangentes sobre os cuidados médicos de seus bebês, oportunidades de participar da tomada de decisões e envolvimento nas atividades de cuidados com o bebê ^( 8 )^ . 

 As pesquisas sobre o papel dos enfermeiros no ambiente da UTIN ainda são limitadas, incluindo pesquisas sobre a facilitação do vínculo precoce entre mães e bebês prematuros. Estudos de avaliação de necessidades de UTINs ^( 9 )^ identificaram que os enfermeiros neonatais exercem um papel relevante tanto no atendimento às necessidades dos pais, quando seus bebês estão em tratamento intensivo, quanto em relação à saúde dos bebês prematuros. A unidade de terapia intensiva neonatal é um ambiente terapêutico projetado para cuidar de recém-nascidos de alto risco. A condição instável do bebê, os procedimentos arriscados e o assédio verbal dos pais são preocupações enfrentadas pelos enfermeiros da UTIN ^( 10 )^ . Este estudo, portanto, tem como objetivo explorar as experiências dos enfermeiros na facilitação do vínculo entre as mães e seus bebês prematuros na UTIN. 

## Método

### Delineamento do estudo

 Este é um estudo descritivo qualitativo com uma abordagem interpretativa ^( 11 )^ . Essa abordagem interpretativa pode explorar e descrever problemas de pesquisa e resumir um fenômeno de forma abrangente ^( 12 )^ . Além disso, a equipe de pesquisa tem um interesse comum na saúde e no bem-estar de bebês prematuros e é composta por três mulheres especialistas em Enfermagem Pediátrica e um homem especialista em Enfermagem da Família. A equipe de pesquisa não garante um relacionamento especial com nenhum dos participantes deste estudo. 

### Local e participantes

 Foi utilizada a técnica de discussão de grupo focal (GF), seguida de entrevistas aprofundadas para corroborar os resultados. Os dados foram coletados por meio do aplicativo *Zoom,* usando a técnica de GF e sessões de entrevistas presenciais. As atividades de GF foram conduzidas com o pesquisador principal e a enfermeira da família. Além disso, a reunião do GF ocorreu duas vezes. 

 A primeira reunião foi realizada à tarde porque a sua natureza *online* permitiu a participação dos diferentes profissionais que trabalhavam em diferentes turnos (manhã, tarde e noite). Em comum acordo, a segunda reunião também foi realizada à tarde, pois a maioria dos enfermeiros líderes de equipe trabalha no turno da manhã. Todos os enfermeiros participaram da primeira reunião do GF no mesmo dia e horário, usando o aplicativo *Zoom* , apenas dois enfermeiros não participaram da segunda reunião. Embora tenha havido problemas de rede durante as atividades do GF, os participantes responderam a todas as perguntas. 

As entrevistas em profundidade foram conduzidas apenas pelo pesquisador principal. As entrevistas foram realizadas face a face na sala de treinamento da unidade neonatal, imediatamente após o turno da manhã. A codificação foi feita pelo pesquisador principal e os resultados da codificação foram analisados por especialistas em enfermagem pediátrica. A população deste estudo foi composta por enfermeiros que trabalharam na UTIN de setembro a dezembro de 2022.

 Os participantes foram selecionados propositalmente usando amostragem estratégica de casos críticos ^( 13 )^ para maximizar as informações necessárias. Os pesquisadores explicaram os motivos da pesquisa e organizaram as rotinas dos participantes por 2 a 3 horas/dia durante três meses para criar familiaridade com quatro hospitais de referência em uma grande cidade de um país em desenvolvimento. Os hospitais foram selecionados de acordo com os seguintes critérios: (1) hospitais públicos com uma unidade neonatal, (2) hospital acreditado nacionalmente e (3) a unidade neonatal estava implementando o CCF. Além disso, os participantes tinham de atender aos critérios de inclusão, que eram: ter trabalhado na UTIN por pelo menos cinco anos, ser líder de equipe e poder se comunicar *online* . Há apenas dois ou três líderes de equipe em cada UTIN do hospital. Não houve desistências, já que todos os participantes foram cooperativos durante o processo de coleta de dados. 

### Coleta de dados

 O instrumento utilizado neste estudo foi um guia geral de perguntas relacionadas às experiências dos enfermeiros na facilitação do vínculo entre as mães e seus bebês prematuros. As perguntas foram, então, desenvolvidas de acordo com as respostas dos participantes durante o GF e as entrevistas em profundidade. Várias ferramentas de apoio foram mobilizadas durante a coleta de dados, incluindo papéis de carta e folhas de anotações de campo para registrar detalhes importantes, como expressões faciais dos participantes e atitudes em relação aos pesquisadores durante a coleta de dados. No estudo, um gravador de voz foi usado durante as entrevistas em profundidade com as três participantes. O emprego do gravador não foi necessário durante as atividades de GF, pois estas foram gravadas usando o aplicativo de reunião *Zoom* . Os pesquisadores testaram as ferramentas de coleta de dados antes de iniciarem o processo, por meio de Grupos Focais (GFs) com quatro enfermeiros gerentes das salas da UTIN em cada hospital onde o estudo foi realizado, além da realização de entrevistas aprofundadas com dois enfermeiros. Os resultados dos GFs e das entrevistas foram avaliados por especialistas em enfermagem pediátrica para obter um *feedback.*

 A primeira etapa do processo de coleta de dados foi identificar os possíveis participantes com base nos critérios de inclusão definidos pelo pesquisador principal. O gerente da UTIN colaborou com o pesquisador principal informando quais enfermeiros atendiam aos critérios e eram participantes em potencial. O pesquisador principal realizou uma sessão de orientação com os possíveis participantes para criar confiança entre eles ^( 14 )^ . Todos os possíveis participantes se mostraram dispostos a assinar o termo de consentimento depois que o pesquisador principal explicou as metas, os objetivos e os procedimentos a serem seguidos durante a pesquisa. A coleta de dados usando a técnica GF foi realizada *online* em uma sala *Zoom* para facilitar as reuniões com todos os participantes. O método *Zoom* foi usado para gerenciar com eficiência as diferenças de turno entre os participantes. 

As entrevistas em profundidade foram realizadas pessoalmente em uma sala calma e silenciosa, usando a entrevista tipo semiestruturada enquanto se observava as atitudes e expressões faciais dos participantes. As sessões de GF foram realizadas duas vezes, com cada sessão durando de 73 a 82 minutos, já as entrevistas em profundidade duraram de 30 a 40 minutos. A coleta de dados começou em 4 de setembro de 2022 e foi concluída em 5 de dezembro de 2022. A primeira análise foi realizada para o primeiro GF, que contou com a presença de 13 participantes representando quatro hospitais de referência em uma grande cidade, garantindo a integridade das transcrições e a impressão do pesquisador sobre a qualidade dos dados. O anonimato foi implementado durante o processo de análise pelos pesquisadores.

O segundo GF foi realizado para validar os resultados do primeiro GF e para garantir que todos os participantes tivessem respondido. O segundo GF também proporcionou uma oportunidade de fazer perguntas que não haviam sido incluídas durante o primeiro GF. A análise dos dados foi, então, realizada com base nos resultados das entrevistas aprofundadas realizadas com os participantes do GF que trabalhavam na UTIN há mais tempo. O processo de entrevista foi registrado com um gravador de voz.

 O pesquisador principal garantiu a integridade das transcrições, da documentação, das anotações diárias e da qualidade dos dados. Um membro da equipe de pesquisa realizou a transcrição e o registro de todos os dados demográficos da pesquisa. Todos os dados foram armazenados no *Google Drive* e só puderam ser acessados pela equipe de pesquisa para garantir a segurança dos mesmos. As citações foram identificadas por meio da análise dos resultados das entrevistas em profundidade com os especialistas da equipe de pesquisa. 

No processo de codificação, quando o pesquisador não encontrou nenhuma nova codificação na segunda entrevista, passou para a terceira para confirmação. Assim, o número total de participantes nas entrevistas em profundidade foi 3, dentre os 13 participantes envolvidos nas atividades de GF. O método de auditoria de investigação foi usado pela equipe de pesquisa durante todo o processo de pesquisa, inclusive durante a revisão dos resultados. Além disso, os pesquisadores compararam as observações, os resultados dos GFs e os das entrevistas em profundidade.

### Análise dos dados

 O *Microsoft Word* foi usado para transcrever os resultados dos GFs e das entrevistas, e os resultados foram analisados por meio da análise qualitativa de conteúdo ^( 15 )^ . Dessa forma, o processo de análise de dados começou com a inserção dos resultados da transcrição, das notas de campo e das fotografias, que mostravam as enfermeiras educando as mães dos bebês, no aplicativo de *software* ATLAS.ti, versão 8.4.16, para, então, ajudar os pesquisadores a interpretar e desenvolver conceitos ou hipóteses. 

A análise continuou com as observações das fotos para encontrar um olhar empático no rosto das enfermeiras e com as leituras da transcrição inteira, realizadas de quatro a seis vezes para encontrar declarações significativas que pudessem descrever o conteúdo da pesquisa.

 O pesquisador principal e os codificadores especialistas em enfermagem da família marcaram cada declaração significativa e identificaram os códigos até que nenhum novo código fosse encontrado. Em seguida, o pesquisador principal agrupou os códigos em categorias, identificou as relações entre eles e os temas e desenvolveu conceitos ou hipóteses, orientados por dois pesquisadores especialistas em enfermagem pediátrica. A etapa final do processo de análise de dados foi a verificação das informações dos participantes por meio de outra reunião do *Zoom* . Além disso, a equipe de pesquisa escreveu o estudo usando a lista de verificação *Consolidated Criteria for Reporting Qualitative Research* (COREQ) para descrever os procedimentos usados ^( 16 )^ . 

### Considerações éticas

A aprovação ética foi obtida pelo Comitê de Ética em Pesquisa em Saúde de cada hospital (A, B, C, D) com os números 1075/EC/KEPK-RSDK/2022, B/3233/070/10/2022, 420/10497 e 175 EC/KEPK/2021. Todos os participantes colaboraram com este estudo sem pressão ou coerção. O termo de consentimento livre e esclarecido explicou os objetivos, benefícios e riscos do estudo. O pesquisador manteve a confidencialidade usando apenas as iniciais “P” seguido da numeração para os participantes durante todo o processo de análise de dados, inclusive nas transcrições e nas anotações de campo. As iniciais de código foram P1 para o primeiro participante, P2 para o segundo participante, e assim sucessivamente.

## Resultados

 Um total de 13 enfermeiras neonatais atendeu aos critérios de inclusão e participou deste estudo. As características dos participantes estão apresentadas na Figura 1 . 

**Figura 1 -:** Características dos participantes. Semarang, Indonésia, 2022

 A Figura 1 mostra que todos os participantes eram do sexo feminino, casadas, muçulmanas e javanesas; 46,2% tinham concluído o bacharelado em enfermagem e eram funcionárias públicas (53,8%). A média de idade das participantes era de 36,08 anos e o tempo de trabalho na UTIN de 9,69 anos. 

 Os pesquisadores estabeleceram três temas e 10 subtemas e as respectivas unidade de significado ( Figura 2 ), a partir dos resultados da pesquisa. 

 Conforme a Figura 2 , a facilitação do vínculo mãe-bebê é descrita como as práticas de enfermagem realizadas com a mãe, com os familiares e com as próprias enfermeiras. Na intervenção com foco na mãe, as participantes desempenharam um papel ativo no processo de facilitação. A interação foi melhorada usando uma intervenção que se concentrou em estabilizar a condição das mães antes de elas conhecerem seus bebês, incentivando-as a tocar fisicamente seus bebês, fornecendo educação repetida, acompanhando-as quando visitavam seus bebês na UTIN e fornecendo massagens de lactação. Tais intervenções são descritas nos relatos abaixo. 

**Figura 2 -:** Temas, subtemas e unidades de significado. Semarang, Indonésia, 2022

*UTIN = Unidade de Terapia Intensiva Neonatal

As participantes expressaram que a estabilização da condição da mãe é o primeiro passo para prepará-la física e psicologicamente para interagir de forma ideal com o bebê. Abaixo estão alguns dos trechos que indicam tais aspectos:

*Precisamos estabilizar a condição delas antes de ensiná-las a cuidar de seus bebês prematuros, para que elas possam se sentir mais próximas de seus bebês*

(P4).

*As mães precisam estar fisicamente saudáveis primeiro, não podemos ajudá-las a criar laços se elas ainda estiverem fisicamente doentes. Portanto, temos de nos certificar de que elas estejam saudáveis antes de ensiná-las sobre nutrição, mobilização, para que possam interagir com o bebê*

(P8).

*A preparação psicológica também é importante, pois, como dissemos antes, algumas mães ainda sentem medo e ansiedade, a ponto de terem medo de tocar o bebê, por isso temos de educá-las no início*

(P2).

As participantes também incentivaram as mães que pareciam relutantes em tocar seus bebês, conforme pode ser observado, a seguir:

*Facilitamos para que as mães toquem seus bebês quando elas vêm nos visitar, mesmo que não possam amamentá-los diretamente*

(P4).

*Precisamos aumentar a confiança delas e assegurar-lhes que não há problema em tocar o bebê e que o toque da mãe é muito importante para o bebê*

(P3).

*Outras participantes mencionaram que outra intervenção comum era o treinamento repetido, a exemplo dos seguintes trechos:*

*Precisamos ser mais ativas ou mais frequentes na educação das mães*

(P11).

Precisamos dar educação repetidamente, precisamos criar confiança para que elas permaneçam confiantes e não tenham medo quando pelo menos segurarem o bebê

(P6).

A próxima intervenção das participantes foi acompanhar a mãe quando ela visitou o bebê na UTIN, de acordo com o relatado:

*Temos de acompanhar a mãe durante cada visita, temos de aumentar sua confiança e convencê-la de que não há problema em segurar o bebê e que o toque da mãe é muito importante para o bebê...*

(P5).

*Observamos e acompanhamos por um tempo até que a mãe e o bebê se sintam confortáveis*

(P2).

Outra intervenção mencionada pelas participantes foi a massagem para amamentação, quando necessária. Abaixo estão alguns dos relatos:

*Houve casos em que as mães receberam massagem de lactação e medicação adicional, mas não conseguiram produzir leite materno*

(P1).

*Para a dor causada pelo ingurgitamento, geralmente fornecemos cuidados com a mama fazendo uma massagem de lactação para ajudar a mãe a produzir leite e aliviar o ingurgitamento*

(P11).

O cuidado centrado na mãe (subtema 1) ilustra que a facilitação do vínculo mãe-bebê exige a disposição da mãe em aceitar o nascimento pré-termo. Em se tratando de cuidados com a família, as intervenções que se concentraram nos membros da família envolveram outros membros da família nos cuidados com o bebê prematuro, conforme reportado:

*Como o cuidado com o bebê segue o princípio do cuidado centrado na família, outros membros da família também precisam ser treinados*

(P7).

*Se a mãe estiver cansada ou não estiver bem descansada, outro membro da família pode assumir o controle e ajudar. Portanto, não treinamos apenas o pai ou a mãe, mas também outros membros da família que moram na mesma casa que o paciente*

(P13).

Envolver membros da família, especialmente os que moram na mesma casa, é uma alternativa para algumas participantes.

A terceira intervenção se concentra no cuidador e na melhoria de sua autoestima, segundo o que foi comentado pelas seguintes participantes:

*A confiança do paciente em mim às vezes faz com que eu me sinta valorizada*

(P5).

*Isso nos motiva a acompanhá-los e apoiá-los, tanto com palavras quanto com orações*

(P13).

O sentimento valioso descrito motivou as os enfermeiras a educar e a incentivar as mães a ficarem perto de seus bebês. Tal sentimento também evocou a empatia, expressa pelos participantes:

*Quando os educamos, tentamos nos colocar na posição das mães*

(P1).

*Eu me colocaria no lugar das mães*

(P7).

Além disso, as enfermeiras também tentaram se aproximar das mães e ser proativas, conforme reportado:

*O que fizemos até agora foram abordagens para convencer a mãe de que nada de ruim acontecerá, desde que seja feito corretamente*

(P5).

*Temos de abordá-las para que elas permaneçam confiantes e não sintam medo, pelo menos quando seguram o bebê*

(P9).

No segundo tema, surgiram quatro subtemas que frequentemente evocaram palavras/termos relacionados ao ambiente hospitalar no cuidado de bebês prematuros, quais sejam: percepções negativas das mães sobre si mesmas; apoio limitado dos membros da família; carga de trabalho dos enfermeiros para outras atividades além dos cuidados aos pacientes e hospitais que não se concentraram em facilitar o vínculo mãe-bebê.

As mães com percepções negativas dificultaram o desenvolvimento de práticas por parte das enfermeiras para a facilitação do vínculo. De acordo com as participantes, as barreiras enfrentadas pelas mães – como estresse, ansiedade, medo, falta de confiança, não querer ver/tocar seus bebês, falta de conhecimento sobre como cuidar de bebês prematuros e não produzir leite materno – também são barreiras para as enfermeiras neonatais facilitarem o vínculo mãe-bebê:

*A mãe está estressada ou ansiosa porque ainda não aceitou o fato de o bebê ser pequeno*

(P1).

*A maioria das mães tem medo de se sentir cansada, ansiosa e insegura. Mesmo que seja apenas para tocar o bebê ou alimentá-lo*

(P2).

*Mesmo para tocar o bebê, ainda precisamos aumentar a confiança das mães para fazer isso*

(P11).

Outro aspecto das percepções negativas das mães expressas pelas participantes foi o medo que elas sentem ao interagir com o bebê prematuro, como se pode ver a seguir:

*O problema seria com a mãe, especialmente se for o primeiro filho, ela geralmente tem medo de segurar o bebê*

(P7).

*Normalmente, as mães de bebês prematuros se sentem assustadas e tristes porque não era hora de ter o bebê e elas veem que o bebê é muito pequeno*

(P8).

As participantes também revelaram que algumas mães não queriam ver seus bebês, especialmente no caso de mães primíparas. Os comentários das participantes foram os seguintes:

*Às vezes, elas não querem ficar perto do bebê ou têm muito medo de visitá-lo, principalmente se o bebê estiver preso a muitos equipamentos*

(P3).

*Às vezes, elas não querem nem olhar para o bebê*

(P5).

A falta de compreensão da mãe sobre saúde também foi mencionada como algo que poderia se tornar um obstáculo para as enfermeiras na facilitação do vínculo mãe-bebê, já que isso tinha um impacto sobre a presença mínima da mãe na sala de cuidados com o bebê, conforme reportado:

*Às vezes, o problema é que a mãe tem pouco conhecimento sobre saúde e tem pais que também não têm esse conhecimento*

(P12).

*Elas podem ter uma forte sensação de ansiedade, medo ou falta de experiência, o que é comum para a maioria das novas mães*

(P9).

Além disso, outro aspecto das percepções negativas das mães foi o medo de não produzir leite suficiente, o que reduz sua confiança em aprender a amamentar de forma ideal. Em última análise, esse problema afetaria o vínculo entre a mãe e o bebê. A seguir, é possível observar exemplos do que as participantes reportaram:

*O leite materno que deveria ser produzido, mas não é, automaticamente fará com que a mãe regrida e não volte ao hospital*

(P6).

*Às vezes, o problema com a amamentação é que as mães pensam demais sobre isso, o que dificulta a produção de leite materno*

(P7).

O próximo fator que dificultou o vínculo entre mãe e filho foi a disponibilidade limitada de membros da família para ajudar as mães, conforme reportado:

*Há outro bebê em casa que precisa de cuidados, por isso não podemos ir ao hospital*

(P13).

*O problema que muitas vezes enfrentamos com bebês de baixo peso ao nascer é que, como suas casas ficam longe do hospital, as mães precisam de ajuda com o transporte, elas não podem ir ao hospital sozinhas, então dependem dos maridos para levá-las, e os maridos nem sempre estão disponíveis*

(P8).

O sentimento de estresse ou ansiedade sentido pela mãe bem como a necessidade de oferecer o melhor cuidado e serviço para seu bebê foi um desafio para as enfermeiras neonatais ao cuidar das pacientes. A situação é ainda mais desafiadora quando as enfermeiras também têm de lidar com os problemas socioeconômicos das pacientes e outras responsabilidades, conforme reportado:

*Por exemplo, não podemos monitorá-las por duas horas inteiras porque temos outro trabalho a fazer*

(P3).

*Às vezes, temos de resolver problemas que não estão relacionados à condição da paciente, como problemas familiares. Isso acontece muito*

(P10).

*Do ponto de vista das enfermeiras, cuidamos de bebês de apenas 900g por um longo período, 100 dias de cuidados custariam 100 milhões de rupias em 2009/2010. Naquela época, havia uma mãe que teve um bebê fora do casamento e sem a bênção dos pais, e esse caso exigiu muita ajuda de nós, enfermeiras, em termos de cuidados e ajuda psicológica*

(P5).

Além disso, o envolvimento prolongado, em que os pesquisadores observam as rotinas diárias dos enfermeiros revela, muitas vezes, problemas de comunicação entre os enfermeiros e os membros da família, levando a protestos sobre o tratamento fornecido. Além disso, o estabelecimento de um vínculo entre mãe e filho exige o envolvimento de várias partes, inclusive do hospital. Muitas participantes mencionaram que o horário limitado de visitas e a infraestrutura incompleta do hospital tiveram um impacto negativo sobre as mães que aguardavam a chegada de seus bebês no hospital:

*A maioria das mães não quer esperar na sala de espera porque não há leito associado à UTIN, e elas geralmente se sentem desconfortáveis agachadas após o parto, por isso preferem descansar em casa... As salas de espera são importantes porque permitem que as mães acompanhem seus bebês*

(P4).

*Limitamos o horário de visitas na UTIN. Na UTIN nível 1, elas podem visitar a cada 3 horas para amamentar o bebê, mas na UTIN ainda limitamos o horário de visita à manhã e à tarde*

(P13).

Os fatores de apoio para mães de bebês prematuros também foram fornecidos pelas participantes para ajudá-las a atingir suas metas de amamentação. Tais fatores consistiram em em internos e externos, sendo os internos o entusiasmo das mães em se envolver ativamente nos cuidados com o bebê, enquanto os externos, o envolvimento do marido no apoio às mães, bem como o apoio das próprias participantes em relação à sua competência como enfermeiras, conforme reportado:

*Se a mãe estiver bem motivada, ficamos feliz. Porque se a mãe estiver feliz, o bebê ficará feliz...*

(P11).

*Graças a Deus, em um caso de bebê com baixo peso ao nascer, o pai pôde levar a esposa ao hospital para o Método Mãe Canguru (MMC). Eles geralmente levam suas esposas ao hospital antes do trabalho e as buscam depois do trabalho, essa foi a nossa experiência na unidade perinatal de alto risco. Assim, o bebê pode receber o leite materno, porque o MMC também permite que o bebê receba o leite materno e, como a mãe está motivada, isso também ajuda o bebê*

(P1).

*Se a mãe produz pouco leite materno, nós a ensinamos a extrair mais*

(P12).

*Se o bebê não precisa de oxigênio, se ele pode se alimentar por via oral e seu choro é forte, podemos ensiná-lo a beber e usar o método canguru*

(P7).

*Precisamos ser capazes de motivá-las*

(P9).

Todas as participantes disseram que as mães precisam ter as habilidades necessárias para aplicar um dos princípios do cuidado neonatal de alto risco, o CCF, para que possam se acostumar a cuidar de bebês prematuros em casa. O uso do CCF permite que as mães se envolvam ativamente no cuidado de seus bebês prematuros. De acordo com a declaração das enfermeiras, quando o pesquisador fez a verificação dos membros com todos os participantes, as enfermeiras disseram que precisavam de algumas habilidades relacionadas ao CCF, incluindo o gerenciamento da lactação, o Método Mãe Canguru (MMC) e as habilidades especiais para melhorar o vínculo entre a mãe e o bebê. Nem todas as enfermeiras dos quatro hospitais receberam treinamento em manejo da lactação, mas quase todas receberam treinamento em MMC, entretanto, continuava sendo difícil implementá-lo na ausência de membros da família.

O MMC geralmente era feito ao lado da incubadora dos bebês e as mães o faziam com mais frequência porque não havia horário de visita para os pais, apenas um hospital tinha horário de visita para os pais, e somente por 15 minutos por dia.

**Figura 3 -:** Percepções das enfermeiras sobre a facilitação do vínculo entre mães e bebês prematuros na unidade neonatal

 Os temas e subtemas estão integrados na Figura 3 , a qual mostra os muitos fatores que influenciam o vínculo entre as mães e seus bebês. Na primeira faixa, tem-se os bebês prematuros, com a cor branca descrevendo a sacralidade. Na segunda faixa, mostra-se as mães, com a cor rosa descrevendo a compaixão entre mãe e bebê. Na terceira faixa encontram-se os membros da família, com a cor azul claro, descrevendo a força da mãe e dos bebês prematuros. Na quarta faixa, apresentam-se os enfermeiros, com a cor verde, que descreve o bem-estar, de modo a remeter os enfermeiros como sujeitos que contribuem para a melhora dos quadros dos bebês, das mães e, consequentemente, das famílias. Na última faixa, o hospital aparece com a cor azul escuro, a qual descreve a tristeza na percepção da mãe. 

## Discussão

O primeiro tema nos mostra em detalhes o que as enfermeiras fizeram para facilitar o vínculo na UTIN. No primeiro subtema, a enfermeira se concentra no cuidado com a mãe do bebê. Por meio do debate entre pares com o especialista em neonatologia e o especialista em saúde mental, interpretamos que a estabilização da mãe do bebê é uma ação crucial dos enfermeiros na primeira etapa da facilitação do vínculo.

 O segundo subtema também mostrou que as intervenções de assistência à família com o objetivo de facilitar o vínculo mãe-bebê podem ser realizadas por meio de processos de tranquilização do bebê, o que inclui proporcionar conforto, toque e comunicação enquanto o bebê está na incubadora ^( 17 )^ . O envolvimento dos membros da família também melhora a comunicação entre a equipe de saúde e a família, para, assim, reduzir a ansiedade vivida pelos familiares enquanto esperam o bebê na UTIN ^( 18 )^ . 

 No terceiro subtema, as enfermeiras descreveram que estavam motivadas. Tal fator foi útil para enfrentar diferentes desafios, considerando que há pelo menos três perspectivas dos enfermeiros indonésios na preparação do planejamento da alta: barreiras internas, barreiras externas e diversidade no planejamento da alta ^( 19 )^ . As enfermeiras enfrentaram esses desafios com uma atitude empática e tentaram se colocar na posição das mães, principalmente na educação. 

 Neste estudo, os fatores de barreira foram preenchidos pela mãe, pelo membro da família, pela enfermeira e pelo hospital. Para verificar esse resultado, realizou-se uma auditoria de investigação entre a equipe de pesquisa. Com base em nossas crenças, a infraestrutura hospitalar foi o principal problema que precisou ser modificado pela política de saúde do hospital para apoiar os enfermeiros na facilitação do vínculo ^( 2^ , ^20 - 21 )^ . Algumas participantes expressaram que não há leito para um membro da família enquanto espera pelo bebê. Além disso, não há horário de visita para os pais na UTIN e os problemas socioeconômicos frequentemente enfrentados estão relacionados à infraestrutura hospitalar, especialmente a distância do hospital em relação à casa da mãe. O uso de webcams poderia ser uma solução ^( 22 )^ mas ainda desconhecida nos países em desenvolvimento, sendo necessário verificar sua eficácia quando aplicado em países inseridos nesse mesmo contexto. 

 Os fatores de apoio incluídos neste estudo são: a mãe, a família e as enfermeiras e a competência da enfermeira pode ser um fator importante para influenciar a mãe e o membro da família. Os enfermeiros precisam ensinar a criar vínculos e a interagir com o bebê ^( 10 )^ , melhorar a sensibilidade dos pais ^( 23 )^ , ajudar a mãe ou outro membro da família a ter contato pele a pele com o bebê ^( 24^ - ^25 )^ , proporcionar uma comunicação eficaz ^( 8^ , ^26 )^ , oferecer aconselhamento de apoio ^( 27 )^ , melhorar os cuidados de enfermagem, por exemplo, por meio da musicoterapia ^( 28 )^ , assim como garantir que as intervenções sejam aplicáveis de acordo com a cultura do hospital ^( 29 )^ . Como as enfermeiras reportaram que há uma carga de trabalho, se a intervenção não for aplicável, isso sobrecarregará as próprias enfermeiras. 

 Os três temas que emergiram deste estudo poderiam responder ao objetivo geral desta pesquisa, que era obter uma compreensão profunda das experiências dos enfermeiros na facilitação do vínculo entre mães e bebês prematuros. A resposta positiva dos enfermeiros ao acompanharem mães com bebês prematuros pode ser um ponto forte para que eles ofereçam o melhor cuidado para as mães e seus bebês. Os enfermeiros precisam controlar suas emoções quando estão com as mães e suas famílias. A afinidade pode interferir no sucesso do processo de enfermagem. Além disso, o treinamento para enfermeiros é importante para reduzir o estresse no trabalho em UTINs de nível II e para aumentar o profissionalismo e a empatia no trabalho ^( 30 )^ . 

 É importante que uma enfermeira neonatal desenvolva empatia, pois parte de sua função é fazer a ponte entre a mãe e o bebê. Portanto, as enfermeiras neonatais precisam entender os sentimentos e as emoções que podem ser vivenciados pelas mães de bebês prematuros ^( 4 )^ . Todas as participantes deste estudo concordaram que as intervenções de enfermagem não podem se concentrar apenas no bebê, muito menos no vínculo mãe-bebê. As intervenções de enfermagem precisam ser abrangentes e abordar todo o ambiente externo que cria o vínculo mãe-bebê, inclusive o profissional de enfermagem, que desempenha um papel importante na facilitação da interação mãe-bebê. 

 Os esforços dos enfermeiros neonatais para enfrentar essas barreiras são importantes na UTIN ^( 31 )^ . Ouvir, confiar e compartilhar conhecimento são esforços a serem feitos durante a mentoria. Um relacionamento bem-sucedido entre enfermeiras neonatais e mães pode ser alcançado por meio do compartilhamento de conhecimento, desenvolvimento de competência e negociação. Além disso, o envolvimento do pai como um sistema de apoio para a mãe também é um fator importante que pode ajudar os enfermeiros a superar várias barreiras no apoio às mães de bebês prematuros. Estudos também mostram que os enfermeiros são muito positivos em relação ao envolvimento dos pais ^( 32 )^ . 

 Os pais desempenham um papel importante durante toda a vida do bebê prematuro e têm um papel importante, direto e indireto, no apoio ao desenvolvimento do bebê na UTIN. Os pais podem melhorar diretamente o desenvolvimento e a saúde do bebê promovendo o método canguru, estimulação tátil, estimulação auditiva e estimulação olfativa ^( 25 )^ . As formas indiretas de apoio aos bebês foram feitas pelos pais por meio da aliança de coparentalidade e do apoio financeiro à família. Em termos de formas indiretas, pode-se dizer que o pai influencia indiretamente o desenvolvimento da criança por meio do relacionamento do casal ^( 33 )^ . Isso sugere que os pais precisam ser envolvidos em programas que apoiem as interações entre mãe e filho. 

 Este estudo sugere que um fator que apoia a ligação entre mãe e bebê é o ambiente imediato do bebê, que consiste na mãe, nos cuidadores e nos membros da família. “O empoderamento dos pais é o passo certo para que eles se sintam capazes de desenvolver suas funções como mães ou pais, sendo que um dos empoderamentos dos pais é o apoio emocional” ^( 34 )^ . Os pais receberão esse apoio emocional de forma eficaz se uma enfermeira puder apoiar a mãe, desenvolvendo sua competência em habilidades de cuidado, fornecendo informações e empatia, que podem ser formadas a partir das práticas espirituais da enfermeira. 

 A espiritualidade é uma característica de domínio que permite que as pessoas encontrem propósito e significado na vida ^( 35 )^ . Os pais geralmente encontram significado espiritual em suas próprias vidas ao se concentrarem na vida de seus filhos. Portanto, receber um bebê prematuro na UTIN pode ser uma experiência traumática para os pais. Os enfermeiros que conseguem reconhecer a existência desse significado espiritual podem sentir empatia e facilitar a interação dos pais com seus bebês, de forma que se destaquem em seu papel de pais porque a empatia é uma forma de compaixão, isto é, outra característica relacionada associada à espiritualidade pessoal bem desenvolvida ^( 36 )^ . Este estudo tem a limitação de não considerar um sistema de saúde em uma perspectiva geográfica e cultural específica. Neste estudo, nossas participantes eram do sexo feminino e muçulmanas. Não tivemos participantes do sexo masculino ou de outras origens religiosas. Portanto, os resultados deste estudo podem não refletir a prática de enfermagem na visão dos enfermeiros do sexo masculino e, especialmente, a intervenção focada na mãe pode não refletir as formas de intervenção em outras religiões. 

## Conclusão

Do ponto de vista das enfermeiras deste estudo, os esforços para facilitar o vínculo mãe-bebê podem ser bem implementados se os enfermeiros tiverem habilidades suficientes e contarem com o apoio de uma infraestrutura hospitalar adequada para deixar as mães confortáveis enquanto esperam seus bebês prematuros. Outro aspecto importante é o apoio de membros da família, dos maridos e de pessoas ao redor da mãe para motivá-la e aumentar sua confiança. Os resultados deste estudo podem formar a base para uma compilação de intervenções que podem ser implementadas em unidades neonatais, concentrando-se nos esforços de ligação entre as mães e seus bebês prematuros. Os resultados deste estudo também sugerem a necessidade de procedimentos operacionais padrão e organizados no hospital em relação aos programas de intervenção de enfermagem para facilitar o vínculo mãe-bebê, começando quando a mãe entra na unidade neonatal ou mesmo antes. De acordo com nosso estudo, é importante primeiro estabilizar a saúde mental da mãe para facilitar o vínculo. Portanto, os enfermeiros neonatais precisam entrar em contato com outros enfermeiros de outra unidade, como a ala de parto ou a policlínica obstétrica para garantir que a mãe esteja bem, especialmente se houver risco de parto prematuro.
